# A Notch/IL-21 signaling axis primes bone marrow T cell progenitor expansion

**DOI:** 10.1172/jci.insight.157015

**Published:** 2022-05-09

**Authors:** Kilian Sottoriva, Na Yoon Paik, Zachary White, Thilinie Bandara, Lijian Shao, Teruyuki Sano, Kostandin V. Pajcini

**Affiliations:** 1 Department of Pharmacology and Regenerative Medicine and; 2 Department of Microbiology and Immunology, University of Illinois at Chicago College of Medicine, Chicago, Illinois, USA.

**Keywords:** Hematology, Transplantation, Bone marrow transplantation, T cell development

## Abstract

Long-term impairment in T cell–mediated adaptive immunity is a major clinical obstacle following treatment of blood disorders with hematopoietic stem cell transplantation. Although T cell development in the thymus has been extensively characterized, there are significant gaps in our understanding of prethymic processes that influence early T cell potential. We have uncovered a Notch/IL-21 signaling axis in bone marrow common lymphoid progenitor (CLP) cells. IL-21 receptor expression was driven by Notch activation in CLPs, and in vivo treatment with IL-21 induced Notch-dependent CLP proliferation. Taking advantage of this potentially novel signaling axis, we generated T cell progenitors ex vivo, which improved repopulation of the thymus and peripheral lymphoid organs of mice in an allogeneic transplant model. Importantly, Notch and IL-21 activation were equally effective in the priming and expansion of human cord blood cells toward the T cell fate, confirming the translational potential of the combined treatment.

## Graphical abstract

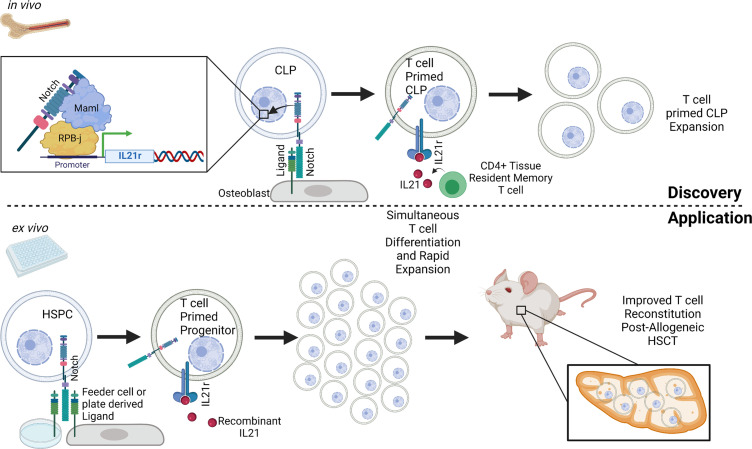

## Introduction

The ex vivo generation of T cell progenitors provides a promising approach for adoptive immunotherapy in patients with diverse hematological ailments (1). Unlike transplantation with mature T cells, allogeneic transplantation with T cell precursors avoids induction of graft-versus-host disease (GVHD), as progenitors undergo selection in the host thymus and are thus restricted by host major histocompatibility complex (MHC) (2). Advances in ex vivo T cell precursor generation have allowed for xenograft-free T cell progenitor production, as well as generation of tumor-specific T cells through introduction of chimeric antigen receptors (3–5). Although human cord blood and induced pluripotent stem cells have been successfully used to generate T cell progenitors ex vivo, advances in the scalability and efficiency of T cell progenitor production are required for clinical translation (6–8). A deeper understanding of the cell signaling pathways involved in early T cell progenitor development is required to improve T cell generation technologies.

The activation of Notch1, which is required for thymopoiesis, on BM-derived progenitors is one of the earliest events in thymic T cell development (9–12). The activation of Notch in early thymocytes leads to stepwise inhibition of alternative lineage pathways, with T cell commitment occurring just prior to β-selection (13). Additional roles for Notch signaling include inhibition of apoptosis and induction of genes required for thymocyte development, including *Gata3*, *Tcf7*, *Bcl11b*, and *Ptcra* (14, 15). Notch1 drives thymocyte differentiation until β-selection, with subsequent development dependent on pre-TCR complex signals (16, 17). The main Notch ligand expressed in the thymus by cortical thymic epithelial cells (TECs) is delta like canonical Notch ligand 4 (Dll4) (18).

The Notch signaling pathway is essential for T cell development and thus is a required component for any T cell production platform. Notch signaling is activated in vivo through binding of cell surface receptors (Notch1–4) with a ligand (Dll1, -3, and -4 and Jagged 1 and 2) expressed by a neighboring cell (19). Ligand interaction induces proteolytic cleavage of Notch, liberating the intracellular domain (ICN), which binds to nuclear recombining binding protein suppressor of hairless (RBPj) and Mastermind-like (MAML) and activates transcription of Notch target genes (20, 21). In mammals, Notch signaling is involved in the development of hematopoietic stem cells (HSCs) and differentiation of several hematopoietic populations, including megakaryocytes, NK cells, and T cells (22–26). Notch signaling is activated ex vivo by coculturing hematopoietic stem and progenitor cells (HSPCs) with Notch ligand–expressing feeder cells or immobilized recombinant ligand (27, 28).

Several recent studies have shown that initiation of the Notch signaling pathway during T lineage development occurs in the BM niche, hinting at a role for Notch in priming progenitors toward a T cell fate before commitment in the thymus (26). Osteocalcin-positive bone cells express Dll4 and Mindbomb, which is required for optimal Notch ligand function, and activate Notch in the common lymphoid progenitor (CLP) population (29, 30). From a cell-autonomous perspective, genetic perturbation of Notch signaling yields a decrease in lymphoid progenitors before the early thymic progenitor population is produced (31). Deletion of either RBPj or GDP-fucose protein O-fucosyltransferase 1 in hematopoietic cells produces a comparable result (29, 32). Additionally, a genetic model for inducible activation and inactivation of RBPj revealed a role for Notch in generating BM lymphoid progenitors by antagonizing the myeloid transcriptional program (33). However, the specific transcriptional pathways activated downstream of Notch in BM lymphoid progenitors are unknown.

Here, we have identified the genes regulated by Notch signaling in the BM CLP population using a recently developed genetic model for Notch perturbation wherein the transcriptional activation domain (TAD) of Notch1 is deleted (25, 31). We found IL-21 receptor (*il21r*) to be a downstream target of Notch signaling in CLPs. The Notch/IL-21r signaling axis drove IL-21–induced CLP proliferation in vivo. Importantly, to our knowledge, IL-21 signaling has never been studied in the context of CLP expansion. We show that the addition of recombinant human IL-21 enabled robust generation of ex vivo functional T cell progenitors.

## Results

### Notch signaling induces T lineage–specific lymphoid development in BM.

**Figure 1:**
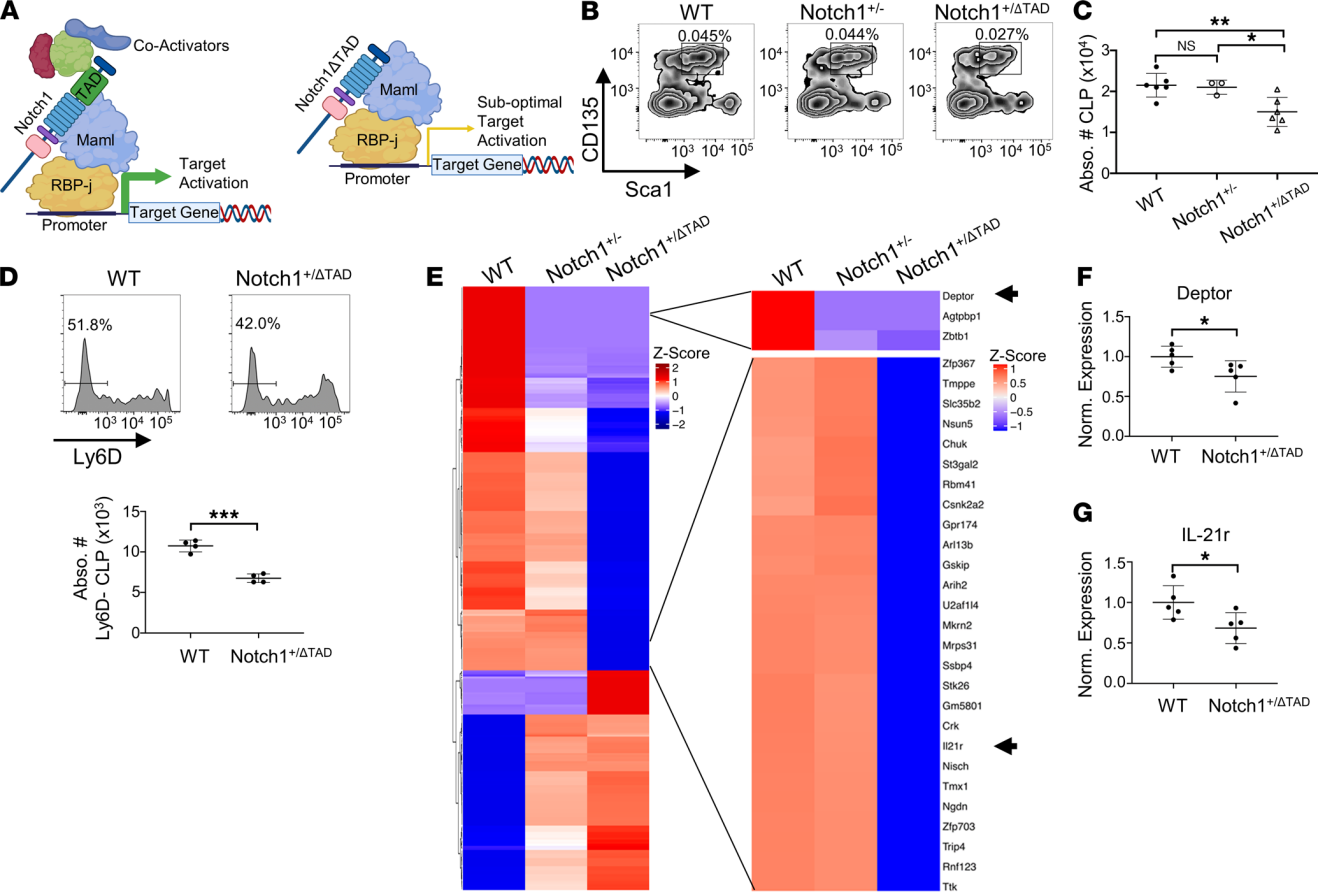
Notch signaling hypomorph reveals CLP defect in the BM. (**A**) Notch1ΔTAD interferes with Notch1 by forming a trimolecular complex with RBPj and MAML but weakly activating transcription without TAD domain–dependent coactivator recruitment. (**B**) Representative flow plot for CLPs (CD135^+^Sca1^lo^), gated on Lin^–^cKit^lo^CD127^+^, with percentage of live cells indicated. (**C**) Absolute number of BM CLP cells in WT (*n* = 6), Notch1^+/–^ (*n* = 3), and Notch1^+/ΔTAD^ (*n* =6). (**D**) (Top) Representative histogram for Ly6D expression on CLPs, gated on Lin^–^cKit^lo^CD127^+^CD135^+^Sca1^lo^. (Bottom) Absolute number of Ly6D^–^ BM CLP cells in WT (*n* = 4) and Notch1^+/ΔTAD^ (*n* = 4). (**E**) Differential gene expression shown as *Z* score heatmap determined by RNA-Seq of mRNA isolated from WT (*n* = 5), Notch1^+/–^ (*n* = 5), and Notch1^+/ΔTAD^ CLPs (*n* = 3). (Right) Select list of differentially expressed genes. (**F**) Expression of Deptor relative to EF1α normalized to WT determined by qRT-PCR of mRNA isolated from CLPs sorted from WT (*n* = 5) and Notch1^+/ΔTAD^ (*n* = 5) mice. (**G**) Expression of IL21r relative to EF1α normalized to WT determined by qRT-PCR of mRNA isolated from CLPs sorted from WT (*n* = 5) and Notch1^+/ΔTAD^ (*n* = 5) mice. *P < 0.05, **P < 0.01, ***P < 0.001. Statistical analysis performed using Student’s 2-tailed *t* test (**D**, **F**, and **G**) and 1-way ANOVA (**C**).

To investigate the role of Notch signaling in T cell progenitor production in the BM, we analyzed BM lymphoid progenitor populations using a hypomorphic genetic mouse model in which 1 allele of the TAD of Notch1 is deleted (31). In this model, Notch1ΔTAD competes with the WT allele for RBPj binding sites in the nucleus, thus activating transcription but failing to recruit coactivators necessary for high transcriptional output, as depicted visually (Figure 1A) (25). Notch1^+/ΔTAD^ mice had no differences in the numbers of BM B cell progenitors or NK progenitors (NKPs), Hardy fractions A and B, and the pre-NKPs and refined NKPs (Supplemental Figure 1, A–D; supplemental material available online with this article; https://doi.org/10.1172/jci.insight.157015DS1) (34, 35). In agreement with previous reports, Notch1^+/ΔTAD^ mice had a decrease in the number of Notch-dependent thymic DN3 cells (Supplemental Figure 1, E and F) (31). However, we found no difference in production of mature B cell, NK cell, or T cell populations in the peripheral blood (Supplemental Figure 1, G–N). The lack of peripheral T cell phenotype in Notch1^+/ΔTAD^ mice despite the decrease in DN3 cells can be explained by the wave of Notch-independent thymocyte proliferation before TCRα rearrangement during the DN to double-positive (DP) transition (36). We then examined the BM CLP population. To differentiate between transcriptional defects caused by loss of 1 allele of Notch1 versus effects of Notch1ΔTAD transcriptional interference, we compared the CLP population of WT, Notch1^+/–^, and Notch1^+/ΔTAD^ mice after confirmation of mutant protein expression in magnetically purified CD25^+^ thymocytes (Supplemental Figure 2A). We observed a significant decrease in the numbers of CLPs in the BM of Notch1^+/ΔTAD^ mice compared with WT, and no difference between WT and Notch1^+/–^, suggesting that the CLP phenotype resulted from Notch1ΔTAD-mediated transcriptional interference (Figure 1, A–C). The CLP population gives rise to B cell, NK cell, and T cell progenitors, and Ly6D^–^ CLPs have been shown to have robust T cell lineage potential (26, 29, 37). We thus examined CLP Ly6D expression and found that Notch1^+/ΔTAD^ mice had significantly fewer Ly6D^–^ CLPs in the BM compared with WT mice (Figure 1D). Taken together, these results indicate that production or maintenance of BM CLP cells requires robust Notch activation.

### IL21R is a BM-specific Notch target in the CLP population.

We then set out to identify the transcriptional targets downstream of Notch signaling in BM T lineage progenitors. We sorted CLP cells from the BM of WT, Notch1^+/–^, and Notch1^+/ΔTAD^ mice and isolated RNA for whole-transcriptome RNA-Seq. After alignment and differential expression analysis (DESeq2), we found 299 differentially expressed genes between WT and Notch1^+/ΔTAD^ CLP cells, including canonical Notch target DEP domain containing MTOR interacting protein (Deptor), which was affected in both Notch1^+/–^ and Notch1^+/ΔTAD^ mutants (Figure 1E) (38). Of particular interest was IL21r, which was in a cluster of genes specifically downregulated in Notch1^+/ΔTAD^ CLPs and whose expression was unaffected by loss of 1 allele of Notch1 (Figure 1E). IL-21 is a member of the common cytokine receptor γ chain family of cytokines and is a ligand for the IL21r/IL2rb receptor complex (39). IL-21 signaling has roles in multiple lymphoid processes, such as maintenance and functionality of CD8^+^ T cells, differentiation of plasma cells, and induction of Th cell differentiation (40–46). Furthermore, several distinct loss-of-function mutations in the human IL21r gene have been described in patients with primary immunodeficiency syndrome (47). We confirmed IL21r and Deptor as transcriptionally regulated targets of Notch via quantitative reverse transcription PCR (qRT-PCR) of sorted BM CLPs from WT and Notch1^+/ΔTAD^ mice (Figure 1, F and G).

IL21r has recently been shown by ChIP-Seq to be a direct Notch1 target in human small B cell lymphomas (48). Upon further analysis of this published data set generated in the mantle cell lymphoma cell line Rec-1, we found a consensus RBPj site within intron 2 of the IL21r locus, which showed strong binding of RBPj and MAML, as well as γ-secretase inhibitor–sensitive (GSI-sensitive) Notch1 binding (Supplemental Figure 2B). We subcloned a 470 bp region of human IL21r intron 2 containing the RBPj site or a mutated RBPj site, designed and validated using transcription factor binding prediction software PROMO, into the luciferase reporter plasmid pGL3 and performed a luciferase assay in U2OS cells with ICN1 and a dominant-negative version of Mastermind (dnMAML) (Supplemental Figure 2C) (49, 50). Luciferase output was normalized to Renilla control plasmid, and each condition was normalized for background to an empty vector control. ICN1 efficiently induced luciferase expression from the pGL3-IL21r plasmid, and this expression was Notch dependent, as dnMAML reduced output to the range of empty vector alone and mutation of the RBPj site produced significantly less luciferase activity, providing evidence that this is a site of Notch trimolecular complex functional occupancy in intron 2 of IL21r (Supplemental Figure 2D). Thus, we focused on investigating the Notch/IL21r signaling axis in BM lymphoid progenitor cells.

**Figure 2:**
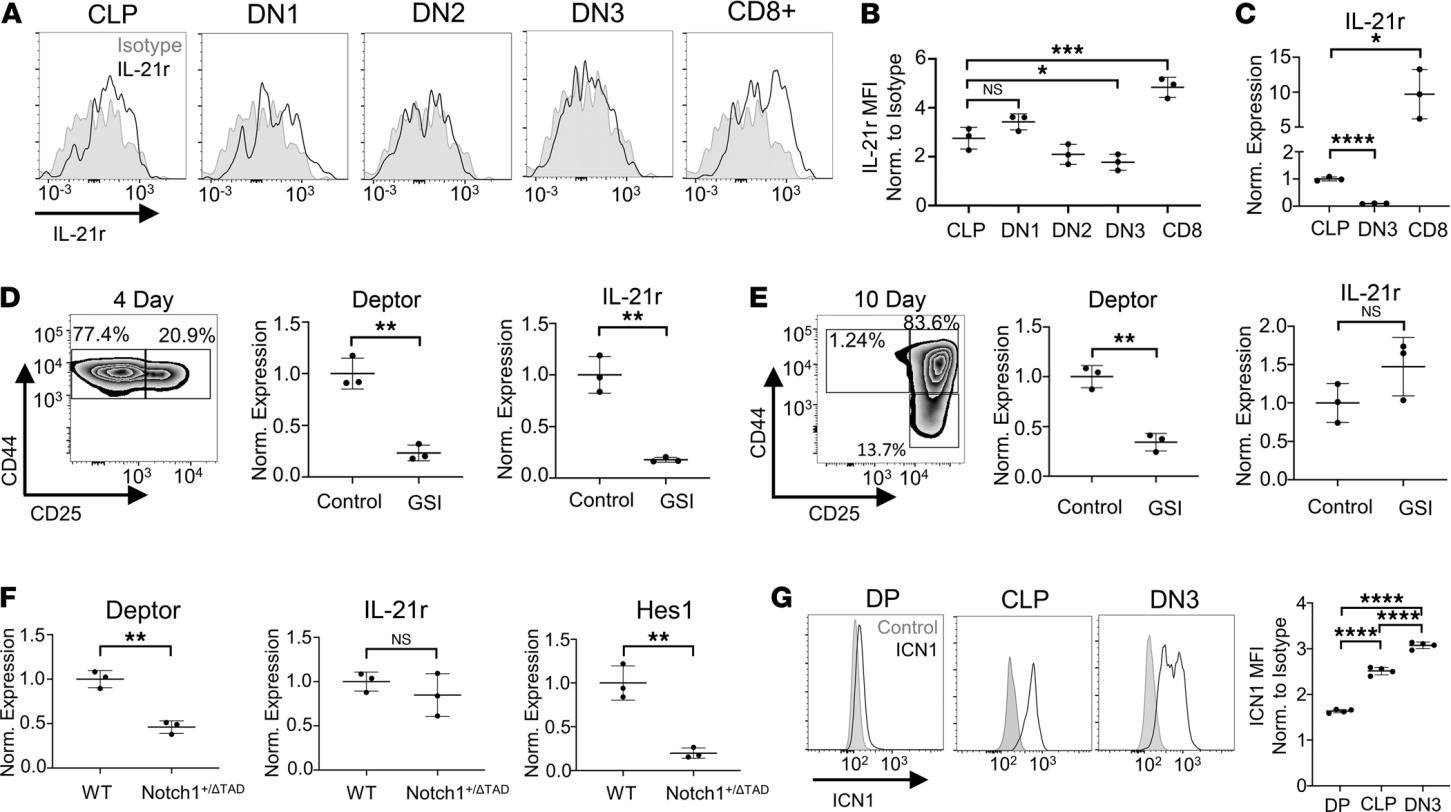
IL21r is a BM-specific Notch target. (**A**) Representative histogram for IL21r staining on BM CLP and thymic DN1, DN2, DN3, and CD8^+^ populations. (**B**) IL21r MFI normalized to isotype control for BM CLP and thymic DN1, DN2, DN3, and CD8^+^ populations (*n* = 3). (**C**) Expression of IL21r relative to GAPDH normalized to CLP determined by qRT-PCR of mRNA isolated from BM CLP and thymic DN3 and CD8^+^ cells from WT (*n* = 3) mice. (**D**) (Left) Representative flow plot of hematopoietic progenitor Lin^–^Sca1^+^cKit^+^ (LSK) cells cultured on OP9-DL1 cells for 4 days, gated on live cells. (Right) Expression of Deptor and IL21r relative to GAPDH normalized to DMSO determined by qRT-PCR of mRNA isolated from DMSO and GSI-treated LSK 4-day cultures (*n* = 3). GSI added for final 24 hours. (**E**) (Left) Representative flow plot of LSK cells cultured on OP9-DL1 cells for 10 days, gated on live cells. (Right) Expression of Deptor and IL21r relative to GAPDH and normalized to DMSO determined by qRT-PCR of mRNA isolated from DMSO and GSI-treated 10-day LSK cultures on OP9-DL1 (*n* = 3). GSI added for final 24 hours. (**F**) Expression of Deptor, IL21r, and Hes1 relative to EF1α normalized to WT determined by qRT-PCR of mRNA isolated from sorted WT or Notch1^+/ΔTAD^ thymic DN3 cells (*n* = 3). (**G**) (Left) Representative histograms for intracellular ICN1 of thymic DP, DN3, and BM CLP populations stained with anti-rabbit–Alexa Fluor 647 secondary antibody. Gray/filled = no primary antibody control, black/empty = ICN1. (Right) MFI of ICN1 normalized to secondary antibody control (*n* = 4). *P < 0.05, **P < 0.01, ***P < 0.001, ****P < 0.0001. Statistical analysis performed using Student’s 2-tailed *t* test (**D**–**F**) and 1-way ANOVA (**B**, **C**, and **G**).

We first examined surface expression of IL21r on CLPs and downstream T cell lineage progenitors via flow cytometry. In agreement with previous work, CD8 single-positive cells expressed the highest levels of surface IL21r in the thymus, with BM CLPs expressing IL21r at a lower level (Figure 2, A and B) (51). Interestingly, although thymic DN3 cells underwent the highest levels of Notch signaling among thymic progenitors, they had less IL21r surface expression than either CLPs or CD8^+^ thymocytes (Figure 2, A and B). This result was confirmed at the transcriptional level via qRT-PCR (Figure 2C). These results suggest that IL21r is transcriptionally regulated by Notch signaling specifically at the CLP level and not in the thymus. To test this, we first sorted BM Lin^–^Sca1^+^cKit^+^ (LSK) cells and cultured them on OP9-Dll1 cells to induce T lineage differentiation ex vivo (52). RNA was isolated after 24 hours of GSI treatment of 4- and 10-day cocultures, at which points ex vivo–cultured progenitors phenotypically resembled thymic DN1 and DN2/DN3 cells, respectively (Figure 2, D and E). qRT-PCR revealed that while Deptor expression was affected by GSI at both 4- and 10-day time points, IL21r expression was specifically downregulated by GSI in the 4-day culture (Figure 2, D and E). We further examined the temporal regulation of IL21r by Notch activation in T cell development in vivo using Notch1^+/ΔTAD^ mice. Consistent with the ex vivo–derived T cell precursors, thymic DN3 cells sorted from Notch1^+/ΔTAD^ mice had lower expression of Deptor than those sorted from WT mice but showed no change in IL21r expression (Figure 2F). Canonical Notch target Hes1 was used as a positive control for Notch1ΔTAD-dependent transcriptional downregulation (Figure 2F). Activation of the Notch signaling pathway was confirmed in these populations in WT mice using intracellular flow cytometry for cleaved, activated Notch1 (Val1744). We found high Notch activity in DN3 cells and increased Notch activity in BM CLP cells compared with thymic DP cells, which did not undergo active Notch signaling (Figure 2G) (9). Finally, we found that IL21r was initially expressed in the lymphoid primed multipotent progenitor population compartment, though at lower levels than in CLPs (Supplemental Figure 2E). Taken together, these results show that Notch-mediated transcriptional activation of IL21r is temporally restricted to BM CLPs during prethymic T cell progenitor development.

### IL-21–induced CLP proliferation is Notch dependent.

**Figure 3:**
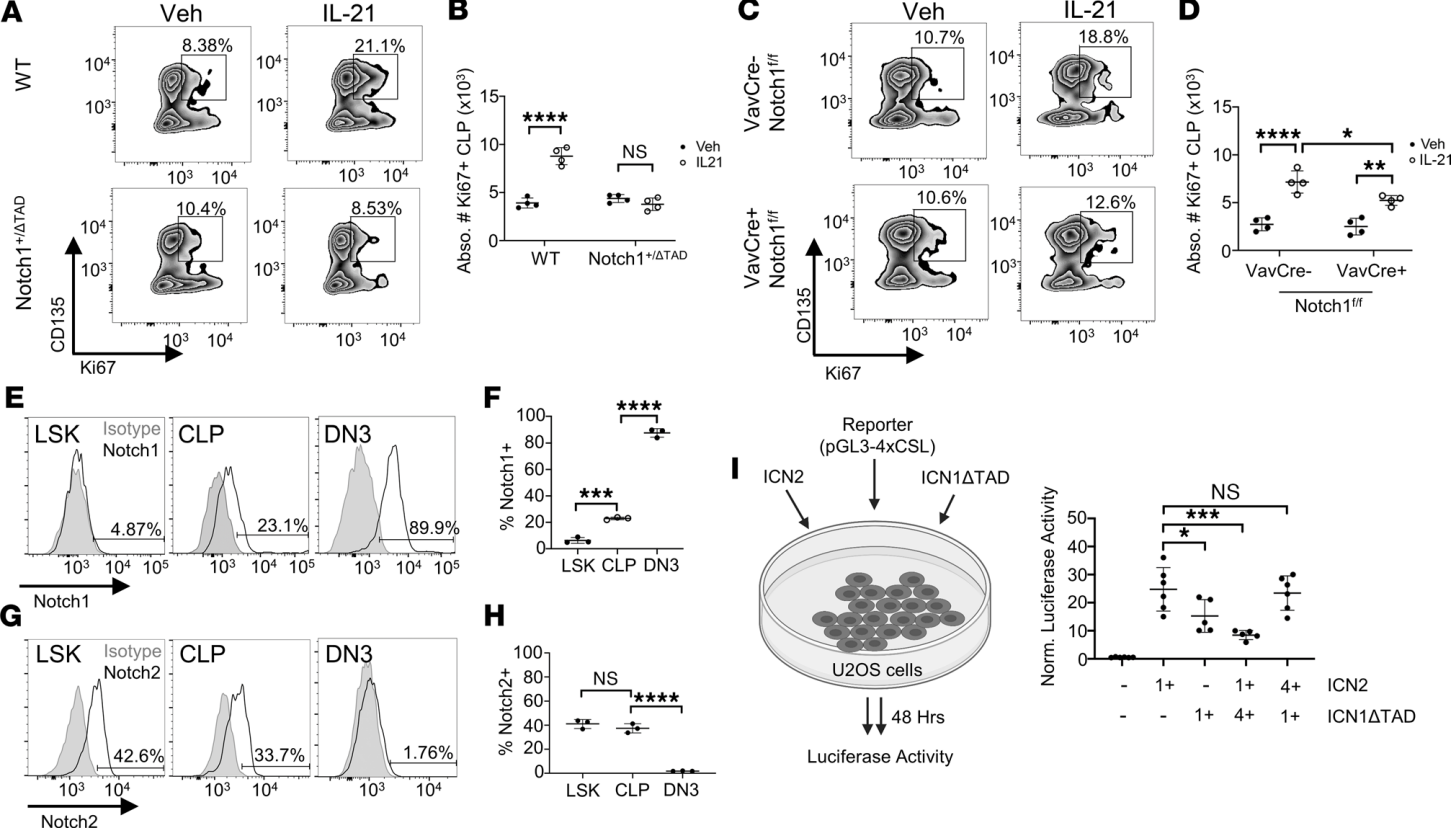
IL-21–induced CLP proliferation is Notch dependent. (**A**) Representative flow plot for Ki67 staining on BM CLP population after vehicle or IL-21 i.p. injections in WT or Notch1^+/ΔTAD^ mice, gated on Lin^–^cKit^lo^Sca1^lo^CD127^+^. (**B**) Absolute number of Ki67^+^ CLP cells in BM of WT or Notch1^+/ΔTAD^ mice after vehicle or IL-21 i.p. injections (*n* = 4). (**C**) Representative flow plot for Ki67 staining on BM CLP population after vehicle or IL-21 i.p. injections in Vav-Cre^–^ Notch1^fl/fl^ or Vav-Cre^+^ Notch1^fl/fl^ mice, gated on Lin^–^cKit^lo^Sca1^lo^CD127^+^. (**D**) Absolute number of Ki67^+^ CLPs in BM of Vav-Cre^–^ Notch1^fl/fl^ or Vav-Cre^+^ Notch1^fl/fl^ mice after vehicle or IL-21 i.p. injections (*n* = 4). (**E**) Representative histograms for Notch1 staining on BM LSK, CLP, and thymic DN3 populations. Gray/filled = isotype control. (**F**) Percentage of BM LSK, CLP, and thymic DN3 populations expressing Notch1 (*n* = 3). (**G**) Representative histogram for Notch2 staining on BM LSK, CLP, and thymic DN3 populations. Gray/filled = isotype control. (**H**) Percentage of BM LSK, CLP, and thymic DN3 populations expressing Notch2 (*n* = 3). (**I**) (Left) U2OS cells were transfected with reporter pGL3-CSL4X, and with empty control vector, ICN2, ICN1ΔTAD, or both ICN2 and ICN1ΔTAD. (Right) Firefly luciferase activity normalized to Renilla activity in U2OS cells transfected with pGL3-CSL4x and empty vector, ICN2, or ICN1ΔTAD at indicated ratios (*n* = 5–6). *P < 0.05, **P < 0.01, ***P < 0.001, ****P < 0.0001. Statistical analysis performed using 1-way ANOVA (**F**, **H**, and **I**) and 2-way ANOVA (**B** and **D**).

We then set out to examine the functional role of the Notch/IL21r axis in the CLP population in vivo. Since IL-21 is a potent inducer of proliferation in lymphoid cells, we examined CLP proliferation in WT and Notch1^+/ΔTAD^ mice after IL-21 treatment. Mice were given 3 injections of 50 μg/kg IL-21 or PBS control 48 hours apart (53). BM was isolated for intracellular Ki67 staining of the CLP population 24 hours after the final injection. Although IL-21 induced a 2-fold increase in the number of Ki67^+^ CLP cells in WT mice, Notch1^+/ΔTAD^ CLPs failed to respond to IL-21–mediated proliferation (Figure 3, A and B). As IL21r expression was unaffected in Notch1^+/–^ mice (Figure 1E), we hypothesized that the failure of Notch1^+/ΔTAD^ CLPs to respond to IL-21 was a Notch1ΔTAD-specific effect. We therefore repeated the IL-21 injection regimen using Notch1^fl/fl^ mice that were crossed with the *vav* panhematopoietic Cre driver strain (54). Vav-Cre expression resulted in deletion of Notch1 and abrogation of Notch-dependent DN3 development (Supplemental Figure 2, F and G). Interestingly, Vav-Cre^+^ Notch1^fl/fl^ CLPs were only partially responsive to IL-21–mediated proliferation, as detected by Ki67 (Figure 3, C and D). Even so, the number of Ki67^+^ CLPs from Vav-Cre^+^ Notch1^fl/fl^ BM was significantly less than in Vav-Cre^–^ Notch1^fl/fl^ CLPs after IL-21 treatment (Figure 3, C and D). Importantly, Vav-Cre expression did not affect homeostatic CLP proliferation (Supplemental Figure 2H). This would suggest that other Notch receptors may play a role in IL-21–induced CLP proliferation.

To further investigate the Notch-dependent transcriptional regulation of IL21r in BM CLPs, we performed surface expression analysis by flow cytometry for individual Notch receptors on CLPs. In agreement with previous work that used Cre reporters to determine Notch receptor expression in individual cell populations, we found that BM CLPs expressed both Notch1 and Notch2 (Figure 3, E–H) (55). Notch2 has been shown to be involved in generation of HSPCs during BM regeneration (56). Our group has previously shown that the Notch1ΔTAD protein interferes with WT Notch1 transcriptional activation in a dose-dependent manner (31). We next examined the effect of Notch1ΔTAD transcriptional interference on Notch2. We transfected U2OS cells with a luciferase reporter plasmid containing 4 consecutive RBPj binding sites along with varied doses of WT ICN2 alone, ICN1ΔTAD alone, or both (Figure 3I). We found that ICN1ΔTAD did indeed interfere with WT ICN2 in a dose-dependent manner (Figure 3I). This, along with the partial response of Vav-Cre^+^ Notch1^fl/fl^ CLPs after IL-21 treatment, reveals the possibility that both Notch1 and Notch2 together drive IL21r transcription to mediate CLP IL-21–induced proliferation.

We next investigated IL-21 as a potential niche factor in the BM CLP niche. As IL-21 is produced mainly by T cells, we first examined IL-21 mRNA production in mature lymphoid populations in the BM (57). We found that CD4^+^ T cells but not CD8^+^ T cells or CD11b^+^, CD19^+^, or NK1.1^+^ cells produced detectible IL-21 mRNA in WT mice (Supplemental Figure 3A). We confirmed this finding using intracellular flow cytometry for IL-21 at the protein level. BM cells were cultured in Brefeldin A–containing media for 5 hours to inhibit secretion and allow for accumulation and detection of cytokines. Our findings show that non-CD4^+^ T cells did not express intracellular IL-21 (Supplemental Figure 3B). We then examined the possibility that Notch/IL-21 signaling plays a role in early lymphoid development in neonatal mice but found that IL-21 mRNA was undetectable in CD3^+^ T cells magnetically purified from the BM of 3-day old WT pups, indicating that any role for the Notch/IL-21 axis is likely limited to older mice with a developed, adaptive immune system (Supplemental Figure 3, C and D).

We then characterized the CD4^+^ T cell population responsible for IL-21 production in the BM niche. We hypothesized that these cells were tissue-resident memory (TRM) T cells, which reside in nonlymphoid tissues and respond to local signaling cues (58). TRM T cells can be defined using surface markers involved in tissue retention, including specific integrin heterodimers, CD44, and CD69, and lack of tissue exit marker CD62L (59, 60). Flow cytometric analysis revealed that nearly all IL-21^+^ cells were CD4^+^CD44^+^ and CD69^+^CD62L^–^, confirming that these cells were probably tissue-resident cells (Supplemental Figure 3, E and F). Furthermore, most of the IL-21^+^ cells also expressed CD29 and CD49d, which make up the integrin heterodimer VLA-4 and are vital for tissue retention of BM hematopoietic populations, including HSPCs (Supplemental Figure 3, E and F) (61). Importantly, the numbers of these cells were similar between WT and Notch1^+/ΔTAD^ mice (Supplemental Figure 3, G and H). Thus, the IL-21–producing cells in the BM are CD4^+^ T cells that display a TRM phenotype.

Although we had confirmed that WT CLP cells are responsive to IL-21–induced proliferation and that CD4^+^ TRM T cells produce IL-21 in WT BM, previous studies had shown that IL-21 signaling does not play a role in lymphoid development under homeostasis (62). We confirmed these findings by examining CLP numbers and Ki67 expression in WT and IL21r-KO mice and found no significant differences (Supplemental Figure 3, I and J). Thus, the responsiveness of CLPs to IL-21–mediated proliferation is not involved during homeostasis, although this does not rule out a role for the Notch/IL21r signaling axis in inflammation or during regeneration.

### IL-21 improves ex vivo T cell progenitor generation.

**Figure 4:**
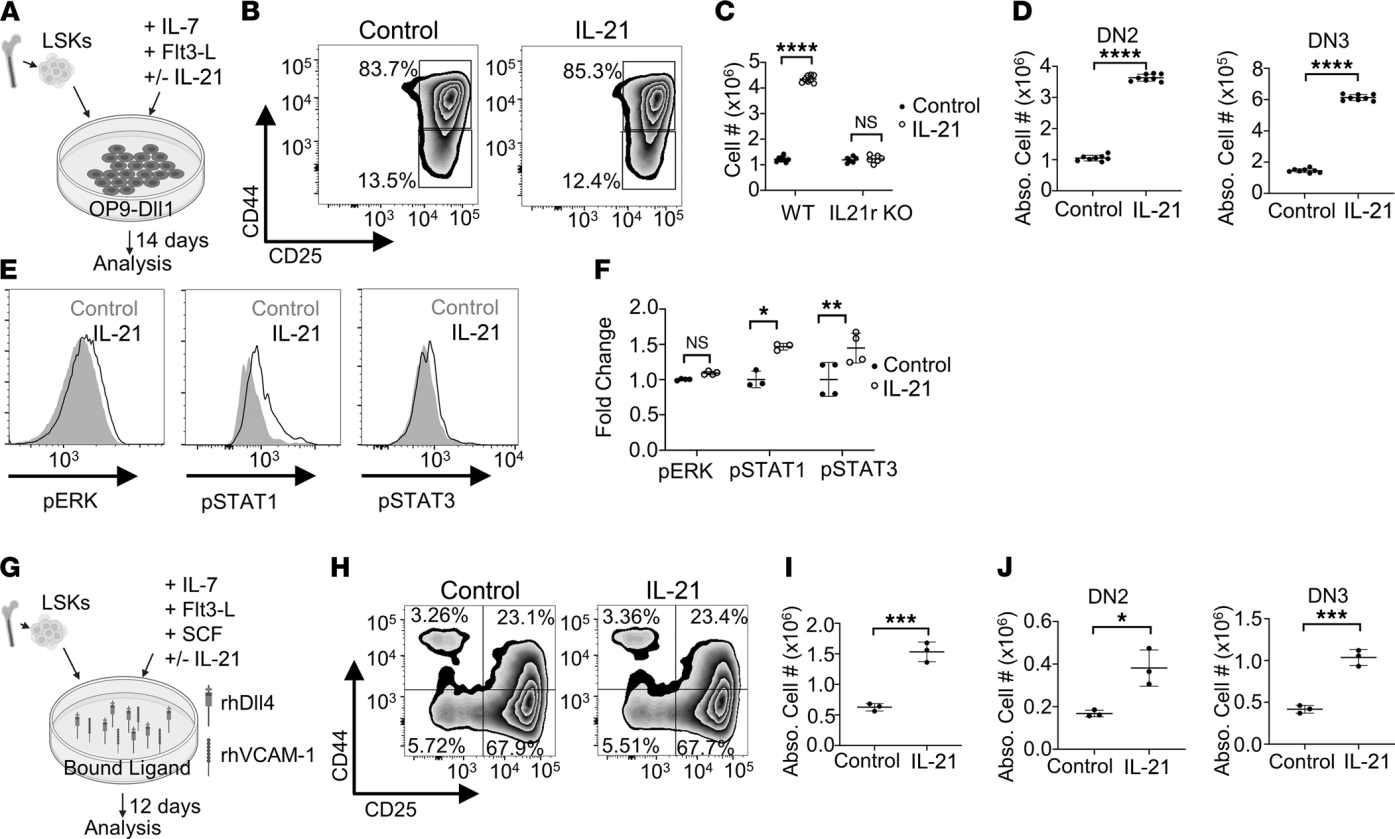
Notch/IL-21 coculture increases production of T cell–primed progenitors. (**A**) Sorted BM LSK cells were cultured 2 weeks on OP9-Dll1 cells with indicated cytokines. (**B**) Representative flow plot for T cell developmental markers on WT LSK cells cultured 2 weeks on OP9-Dll1 cells with or without IL-21, gated on live cells. (**C**) Cell count after 2 weeks’ coculture of WT (*n* = 8) or IL21r-KO (*n* = 6) LSK cells on OP9-Dll1, with or without IL-21. Data generated using individual mice. (**D**) Absolute number of DN2 (*n* = 8) (CD44^+^CD25^+^) and DN3 (*n* = 8) (CD44^–^CD25^+^) cells generated after 2 weeks of OP9-Dll1 coculture, with or without IL-21. Data generated using individual mice. (**E**) Representative histograms of p-Thr202/Tyr204 ERK, p-Ser727 STAT1, and p-Tyr705 STAT3 of 1-week OP9-Dll1 cultured LSK cells after 30-minute treatment of vehicle or IL-21. Gray/filled = control, black/empty = IL-21. (**F**) Fold change in MFI normalized to control for p-Thr202/Tyr204 ERK (*n* = 4), p-Ser727 STAT1 (*n* = 3), and p-Tyr705 STAT3 (*n* = 4) of 1-week OP9-Dll1 cultured LSK cells with or without 30-minute IL-21 treatment. Data generated using individual mice. (**G**) Sorted BM LSK cells were cultured on plates with immobilized rhDll4 and rhVCAM-1 with indicated cytokines. (**H**) Representative flow plot for T cell developmental markers on WT LSK cells cultured 12 days on immobilized rhDll4 and rhVCAM-1 with or without IL-21, gated on live cells. (**I**) Cell count after 12 days’ culture of WT (*n* = 3) sorted LSK cells on immobilized rhDll4 and rhVCAM-1 with or without IL-21. Data generated using individual mice. (**J**) Absolute number of DN2 (CD44^+^CD25^+^) and DN3 (CD44^–^CD25^+^) cells generated after 12 days of culture on immobilized rhDll4 and rhVCAM-1, with or without IL-21. Data generated using individual mice. *P < 0.05, **P < 0.01, ***P < 0.001, ****P < 0.0001. Statistical analysis performed using Student’s 2-tailed *t* test (**D**, **I**, and **J**) and 2-way ANOVA (**C** and **F**). rh, recombinant human.

After characterizing the Notch/IL-21 axis in BM CLPs, we sought to apply our findings to ex vivo T cell progenitor production for the improvement of T cell recovery after HSC transplantation (HSCT). Current ex vivo T cell progenitor–producing protocols use feeder cells that express Notch ligands or purified immobilized Notch ligands along with cytokines necessary for early T cell development, including IL-7 and FMS-like tyrosine kinase 3 ligand (Flt3L) (3, 52). We sorted LSK cells and plated them onto OP9-Dll1 feeder cells in media containing IL-7 and Flt3L, with or without IL-21 (Figure 4A). After 2 weeks in culture, T cell progenitors phenotypically resembled thymic DN2 and DN3 cells based on CD44/CD25 staining, and there was no disruption of differentiation potential by addition of IL-21 (Figure 4B and Supplemental Figure 4A). The starting LSK population was homogenously CD44^+^CD25^–^ (Supplemental Figure 4B). However, IL-21 induced rapid expansion of ex vivo–derived T cell progenitors, with 2-fold and 4-fold increases in cells produced at 1 week and 2 weeks in culture, respectively (Figure 4C and Supplemental Figure 4C). The expansion was IL21r specific, as LSK cells sorted from IL21r-KO mice were not responsive to the IL-21–induced expansion ex vivo (Figure 4C and Supplemental Figure 4C). Thus, the addition of IL-21 to the media yielded ~4-fold more T cell precursors using the same number of starting cells (Figure 4D). The IL-21–induced expansion was mediated by enhanced phosphorylation and activation of phosphorylated (p-) p-Ser727 STAT1 and p-Tyr705 STAT3 but not p-Thr202/Tyr204 ERK (Figure 4, E and F). IL-21 treatment specifically influenced proliferation of T cell progenitors and did not disrupt differentiation, as expression of key T cell lineage transcription factors Bcl11b, Tcf7, and E2A was unchanged by IL-21 addition to the culture conditions (Supplemental Figure 4D) (15). No significant B cell differentiation was induced using this coculture system, with or without IL-21 (Supplemental Figure 4, E and F).

Although IL-21 addition led to rapid T cell progenitor output, the use of murine OP9-Dll1 cells in the culture conditions is not applicable to a clinical setting. Recently, a nonxenogeneic culture system using plate-bound recombinant Dll4 and VCAM-1 has been described for T cell progenitor production from hematopoietic progenitors (3). We adapted this system to induce T cell differentiation of hematopoietic progenitors with or without the addition of IL-21 (Figure 4G). After 12 days in culture, there was no phenotypic difference in T cell progenitor differentiation based on surface staining for CD25 and CD44, as seen by an equal percentage of control or IL-21–treated cells in the DN2 or DN3 quadrants (Figure 4H and Supplemental Figure 4G). Similar to the OP9-Dll1 culture system, we observed a large expansion of T cell progenitors, with a 3-fold increase in cell yield after 12 days (Figure 4I). The addition of IL-21 allowed the production of more ex vivo–derived DN2/DN3 T cell precursors from the same starting number of hematopoietic progenitors (Figure 4J). The levels of myeloid and B cell differentiation using this culture system were negligible, as determined by CD11b and CD19 staining, respectively (Supplemental Figure 4, H and I). Thus, IL-21 improves current ex vivo T cell production platforms by vastly increasing the yield of target cells from small numbers of hematopoietic progenitors.

### Notch–IL-21 ex vivo–generated T cell progenitors produce functional, mature T cells in immunosuppressed hosts.

We next set out to determine the effect of IL-21 treatment on ex vivo–derived T cell progenitors in a transplantation setting. As T cell progenitors were only exposed to IL-21 in culture, during which time no change in the differentiation status was observed, we expected that an equal number of T cell progenitors produced ex vivo in the presence of IL-21 would perform equally as well as non–IL-21–treated cells posttransplant into irradiated hosts. After 2 weeks in culture, we transplanted 1 × 10^6^ ex vivo–derived T cell precursors into sublethally irradiated congenic mice and isolated lymphoid tissues for reconstitution analysis. We found that IL-21–treated cells more efficiently reconstituted the thymus 1 week posttransplant than control non–IL-21–treated cells, even though the same number of cells were initially transplanted in both groups (Supplemental Figure 5, A and B). Similar results were seen in the spleen and lymph nodes 2 weeks posttransplant, with significantly higher engraftment in the spleen and with a trend increase seen in the lymph nodes (Supplemental Figure 5, C–F). The increased engraftment of IL-21–treated cells was not due to increased expression of thymus homing markers CCR7, CCR9, or PSGL1 or signaling receptors involved in T cell differentiation, including Notch1, IL7r, and cKit (Supplemental Figure 5G). Thus, the increased engraftment of IL-21–treated cells is probably due to the rapid proliferation of T cell progenitors (Figure 4C). We additionally performed a limiting dilution assay by transplanting 1 × 10^6^, 0.3 × 10^6^, or 0.1 × 10^6^ ex vivo–derived control or IL-21–treated T cell progenitors into sublethally irradiated congenic recipients. The improved efficiency in thymic reconstitution 1 week posttransplant could be seen when transplanting as few as 0.3 × 10^6^ ex vivo–derived T cell progenitors (Supplemental Figure 5H).

**Figure 5:**
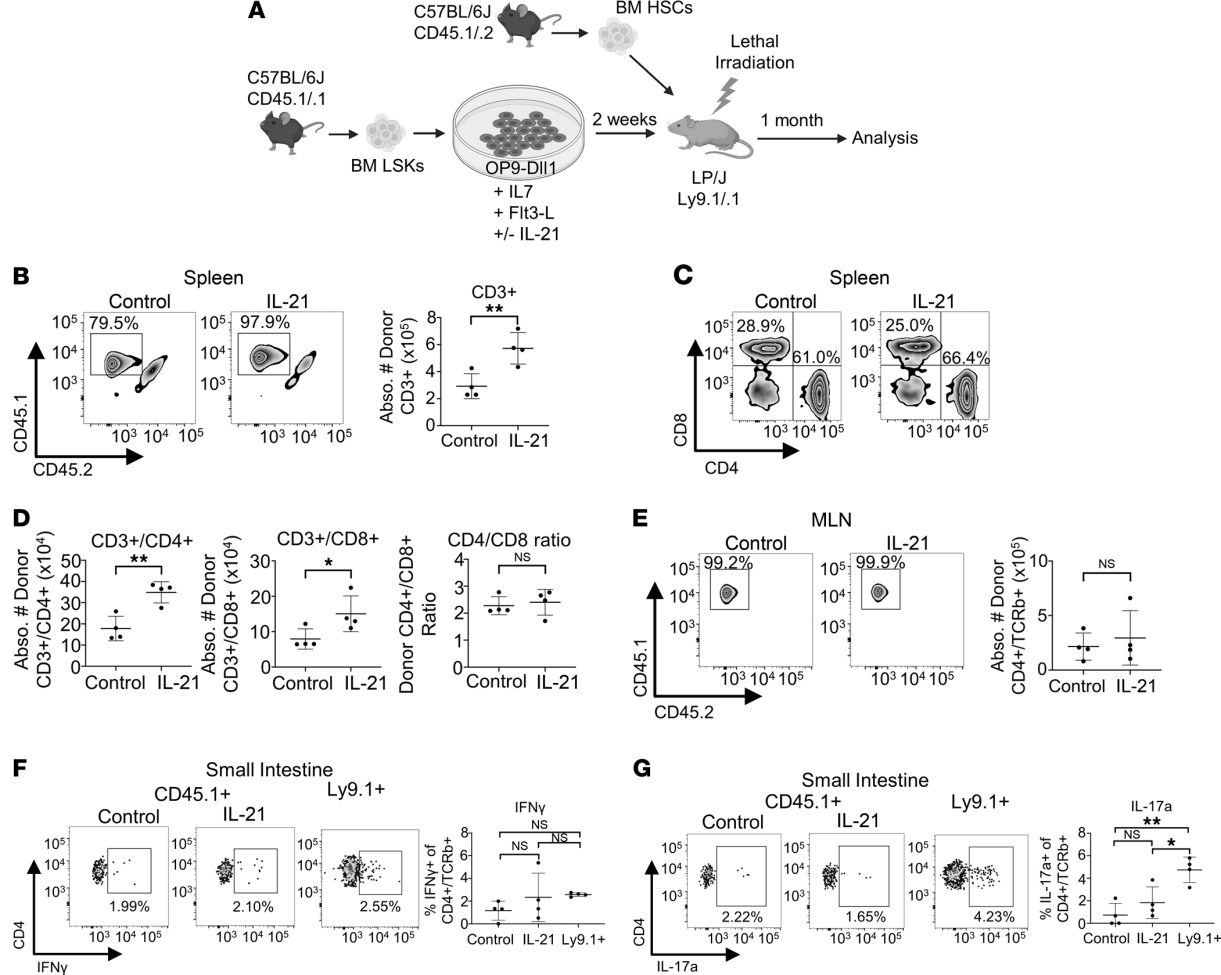
Notch/IL-21–primed T cell progenitors efficiently reconstitute allogeneic recipients. (**A**) Sorted BM LSK cells (CD45.1) were cultured on OP9-Dll1 cells with indicated cytokines. After 2 weeks, 2 × 10^6^ T cell–primed progenitors were cotransplanted with 1000 fresh HSCs (CD45.1/.2) into lethally irradiated LP/J (Ly9.1^+^) recipient mice. (**B**) Representative flow plot (left) and absolute number (right) of donor splenic T cells from control (*n* = 4) and IL-21–treated (*n* = 4) culture recipients, 1 month posttransplant. Gated on Ly9.1^–^CD3^+^. (**C**) Representative flow plot for CD4 and CD8 staining of donor splenic T cells, 1 month posttransplant. Gated on Ly9.1^–^CD3^+^CD45.1^+^CD45.2^–^. (**D**) Absolute number of donor splenic CD4^+^ and CD8^+^ cells and CD4/CD8 ratio of cells from control (*n* = 4) and IL-21–treated (*n* = 4) culture recipients, 1 month posttransplant. (**E**) (Left) Representative flow plot for donor T cells in MLNs, 1 month posttransplant. Gated on MHCII^–^Ly9.1^–^CD4^+^TCRb^+^CD44^+^. (Right) Absolute number of donor T cells in MLNs from control (*n* = 4) and IL-21–treated (*n* = 4) culture recipients. (**F**) (Left) Representative flow plot for IFN-γ of host Ly9.1^+^ and control and IL-21–treated culture (CD45.1^+^) cells from recipient SI, 1 month posttransplant. Gated on MHCII^–^CD4^+^TCRb^+^CD44^+^. (Right) Percentages of T cells expressing IFN-γ of host Ly9.1^+^ (*n* = 4) and control (*n* = 4) and IL-21–treated (*n* = 4) culture (CD45.1^+^) cells from recipient SI, 1 month posttransplant. (**G**) (Left) Representative flow plot for IL-17a of host Ly9.1^+^ and control and IL-21–treated culture (CD45.1^+^) cells from recipient SI, 1 month posttransplant. Gated on MHCII^–^CD4^+^TCRb^+^CD44^+^. (Right) Percentages of T cells expressing IL-17a of host Ly9.1^+^ (*n* = 4) and control (*n* = 4) and IL-21–treated (*n* = 4) culture (CD45.1^+^) cells from recipient SI, 1 month posttransplant. MLN, mesenteric lymph nodes; SI, small intestine. *P < 0.05, **P < 0.01. Statistical analysis performed using Student’s 2-tailed *t* test (**B**–**E**) and 1-way ANOVA (**F** and **G**).

To further assess the functional improvement in T cell reconstitution after transplant of IL-21–expanded ex vivo–derived T cell progenitors, we transplanted C57BL/6J-derived cells into lethally irradiated LP/J recipients. This system (Figure 5A) models allogeneic transplantation in mice (63). In agreement with the congenic transplants, transplantation of an equal number of IL-21–expanded T cell progenitors resulted in significantly improved donor-derived CD3^+^ T cell reconstitution compared with control in the spleen 1 month posttransplant (Figure 5B). Additionally, CD45.1^+^ ex vivo–derived T cell progenitors made up the majority of CD3^+^ T cells in the spleen compared with CD45.1/.2^+^ HSC-derived progenitors, especially in the IL-21 group (>97%) (Figure 5B). This result demonstrates the superiority of ex vivo–derived cells in T lineage reconstitution compared with the slower T cell differentiation kinetics of donor BM HSCs (64). Both the donor-derived CD4^+^ and CD8^+^ T cell populations in the spleen were significantly higher in the IL-21–treated group, with no difference in the splenic CD4/CD8 ratio 1 month posttransplant (Figure 5, C and D). Consistently with other reports, CD45.1^+^ donor-derived T cells in the thymus of either control or IL-21–treated ex vivo T cell progenitor recipients 1 month after HSCT made up a small portion of total thymocytes and were mostly CD4 or CD8 single positive, representing the transient wave of donor-derived thymopoiesis induced by ex vivo–derived T cell progenitors (Supplemental Figure 6, A–C) (63).

To assay the maturation and functional contribution of IL-21–treated ex vivo–derived T cell progenitors posttransplant, we examined donor-derived CD4^+^ T cell populations in the gut. Several CD4^+^ T cell subtypes work together to maintain gut homeostasis, preventing ulcerative colitis and Crohn’s disease in a tissue laden with inflammatory antigens (65, 66). Intestinal effector CD4^+^ T cells, including T-bet^+^ Th1 cells, GATA3^+^ Th2 cells, and RORγt^+^ Th17 cells, respond to intracellular bacteria and viruses by secreting cytokines involved in resolution of gut immune pathology (67, 68). Additionally, regulatory CD4^+^ T cells, including thymus-derived natural Tregs (nTregs) and peripherally derived inducible Tregs (iTregs), limit excessive intestinal inflammation despite the enormous antigen load from the gut by inhibiting effector T cells (69–71).

Despite a trend toward increase, there was no significant difference in the numbers of ex vivo–derived T cells in the mesenteric lymph nodes (MLNs), small intestine, or large intestine between control and IL-21–treated donor cells 1 month posttransplant (Figure 5E and Supplemental Figure 6, D and E). However, in both conditions, almost all (>98%) T cells in each organ were derived from CD45.1^+^ ex vivo–generated T cell progenitors, with no donor HSC-derived mature T cells in the gut (Figure 5E and Supplemental Figure 6, D and E). To further characterize mature T cell subsets, we analyzed T cell transcription factor expression via intracellular flow cytometry. We found no differences in the numbers of Th1 (T-bet^+^), Th2 (GATA3^+^), Th17 (RORγt^+^Foxp3^–^), nTreg (Fox3p^+^RORγt^–^), or iTreg (Fox3p^+^RORγt^+^) in the small or large intestines or MLNs between control and IL-21–treated ex vivo–derived donor cells at 1 month posttransplant (Supplemental Figure 7, A–F). Additionally, there were no differences in the percentages of cells expressing these transcription factors in the large intestines, small intestines, or MLNs, except for the percentage of MLN cells expressing RORγt, which was significantly higher in the IL-21–treated ex vivo–derived donor group (Supplemental Figure 8, A–C). The increased percentage of MLN cells expressing RORγt in the IL-21–treated donor group has been previously suggested by work showing that although IL-21 signaling is dispensable for Th17 development in vivo, IL-21 can induce RORγt expression when combined with TGF-β in vitro (72). Despite the increase in percentage of MLN cells expressing RORγt in the IL-21–treated donor group, there was no significant change in the numbers of any T cell subtype we analyzed in the MLNs, including Th17 (Supplemental Figure 7, A–F, and Supplemental Figure 8C).

We additionally stained for intracellular cytokine production of CD4^+^ T cells in the gut to verify the activity of ex vivo–derived cells 1 month posttransplant. IFN-γ is secreted by effector CD4^+^ T cells mainly in response to microbial antigens, promotes mucus secretion from intestinal secretory cells, and regulates epithelial proliferation and apoptosis (73–75). Control and IL-21–treated ex vivo–derived donor CD4^+^ T cells had no differences in the percentage of cells positive for IFN-γ in the MLNs or small or large intestines of recipient mice (Figure 5F and Supplemental Figure 8, D and E). IL-17a, produced mainly by Th17 cells in the gut, targets epithelial and lymphoid populations to regulate immune reactions to resident microbial populations (76–78). We found no differences in the percentages of either control or IL-21–treated ex vivo–derived donor CD4^+^ T cells expressing IL-17a in the MLNs or small or large intestines 1 month posttransplant (Figure 5G and Supplemental Figure 8, D and E). Additionally, ex vivo–derived donor CD4^+^ T cells in the small intestine produced a comparable amount of IFN-γ compared to Ly9.1^+^ host T cells, and only slightly less IL-17a, confirming that ex vivo–derived T cell progenitors can produce functional mature T cells in vivo (Figure 5, F and G). Taken together, the addition of IL-21 to the media of T cell progenitor ex vivo cocultures improves splenic reconstitution and does not affect the posttransplant ability of these cells to function or differentiate in peripheral organs in an allogeneic setting.

### Human CD34^+^ cord blood cells are responsive to Notch–IL-21–induced T cell progenitor production and expansion.

**Figure 6:**
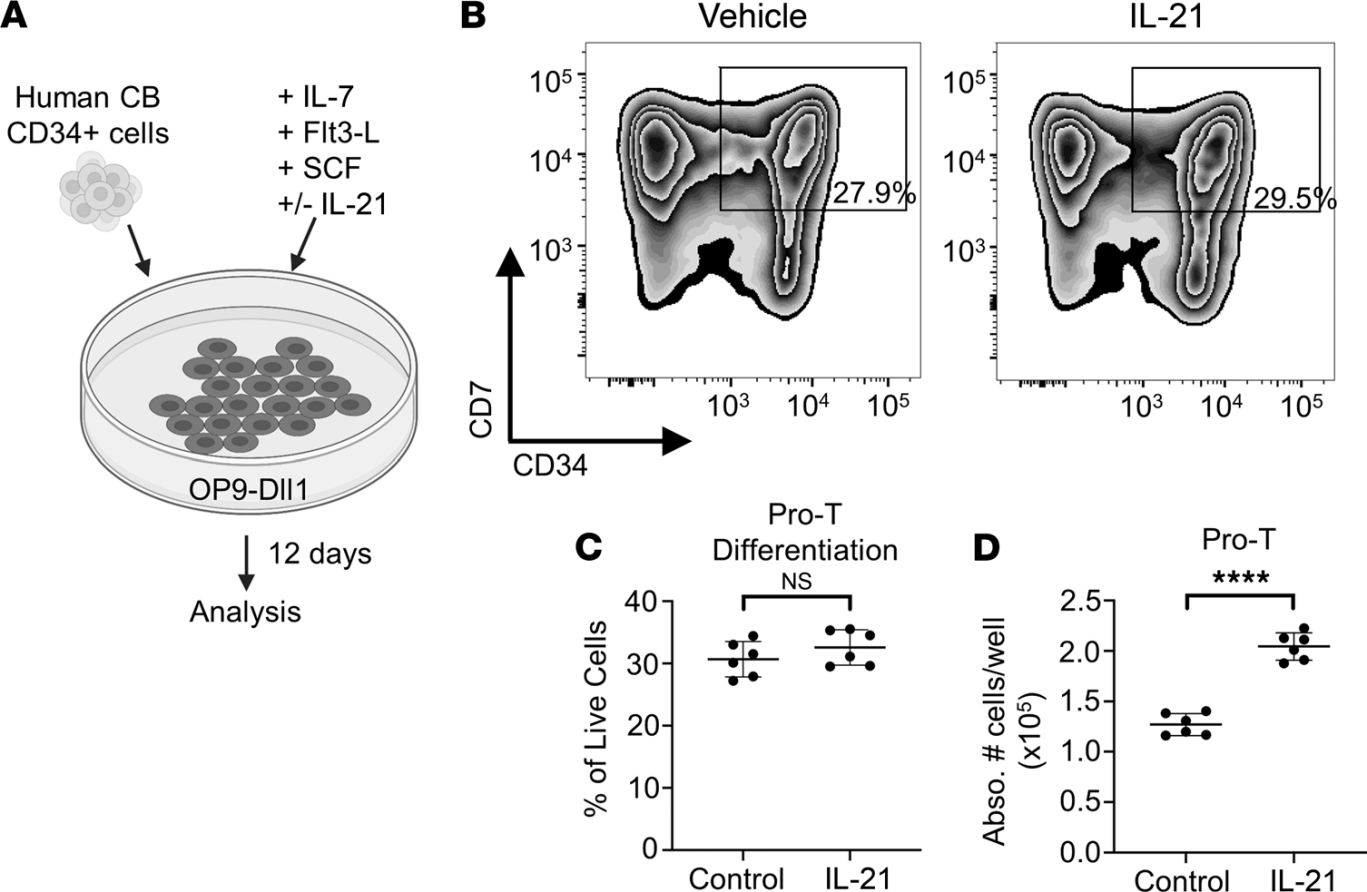
Notch–IL-21 treatment expands human cord blood cells ex vivo. (**A**) Human cord blood (CB) CD34^+^ cells were cultured on OP9-Dll1 cells with the indicated cytokines for 12 days. (**B**) Representative flow plot for human pro–T cell (CD34^+^CD7^+^) markers on human CB cells cultured 12 days on OP9-Dll1 with or without IL-21. Percentage of live cells indicated. (**C**) Quantification of the percentage of CD34^+^CD7^+^ of live CB cells after 12 days’ culture on OP9-Dll1 with or without IL-21 (*n* = 6 from 2 individual donors). (**D**) Absolute number of CD34^+^CD7^+^ cells per well of 12-day-cultured human CB cells on OP9-Dll1 with or without IL-21 (*n* = 6 from 2 individual donors). ****P < 0.0001. Statistical analysis performed using Student’s 2-tailed *t* test (**C** and **D**).

Once we had established that IL-21–expanded T cell progenitors derived from ex vivo culture were functional in an allogeneic transplant model, we turned to human cells to determine if the Notch/IL-21 signaling axis is conserved and can be used toward a similar expansion application. We plated freshly thawed human CD34^+^ cord blood cells on OP9-Dll1 cells in media containing recombinant human IL-7, Flt3L, and stem cell factor, with or without IL-21 (Figure 6A) (79). After 12 days of culture, the percentage of CD34^+^CD7^+^ pro–T cells was equal in the control and IL-21–treated groups (Figure 6, B and C), suggesting no defects in differentiation dynamics. However, we observed significantly more total pro–T cells produced with the Notch–IL-21 combined treatment compared with the Notch-only control, with a 1.5-fold increase in absolute cell numbers (Figure 6D). Thus, Notch–IL-21 combined treatment of human hematopoietic progenitors improves total yield of pro–T cells for potential therapeutic use.

## Discussion

HSCT been a staple for the treatment of blood diseases and disorders since its first successful clinical application in 1957 (80). One of the major clinical consequences of HSCT is the long-term delay in donor-derived T cell recovery (81). Factors such as patient preconditioning, which is necessary to prevent graft rejection by the host immune system, contribute to this delay by damaging organs crucial to lymphoid development, such as the thymus and bone marrow (82–84). The outcome of delayed T cell recovery is defective adaptive immunity, which correlates to an increased incidence of opportunistic infections up to 1 year after HSCT (81, 82, 85). Thus, several strategies have been tested as potential mechanisms for clinical improvement of donor-derived T cell development. Clinical trials directed toward T cell recovery have been focused on improving thymic regeneration and functional integrity (82). Specifically, IL-7 (ClinicalTrials.gov NCT00684008) administration promotes thymocyte survival, while keratinocyte growth factor (NCT01746849) and IL-22 (NCT02406651) provide proliferative and protective signals to TECs (82, 86–90). However, a rate-limiting step in T cell recovery after HSCT remains the seeding of the thymus with BM-derived progenitors (64). This is significant in the setting of transplantation, as thymic seeding induces thymic enlargement and regeneration that ultimately improves the production of HSC-derived T cells (91, 92). Therefore, methods aimed at providing ex vivo–expanded T cell progenitors, which home to the recipient’s thymus and produce mature T cells, provide a promising approach to inducing robust recovery of T cell populations after HSCT (63, 93).

Induction of early T cell differentiation of mouse or human hematopoietic progenitors ex vivo requires activation of Notch signaling commonly provided by feeder cell-derived or plate-bound Notch ligands (52, 92). Cytokines, including IL-7 and Fl3tL, supplement the media ex vivo to promote T cell proliferation, cell survival, and differentiation (94). BM and thymic Notch ligands and IL-7, as well as prethymic Flt3L, are all involved in physiological T cell differentiation and are commonly used in ex vivo T cell differentiation protocols (17). Though they have proved effective to some extent, there is plenty of room for improvement of such protocols, especially as it pertains to integration of novel signaling pathways involved in T cell differentiation, survival, and proliferation. With recent findings indicating a prethymic role for Notch activity in T cell development, the identification of downstream Notch target genes that could be manipulated ex vivo to promote, prime, and expand prethymic T cells represents a critical advance in the area of cell-based therapies (26). We used several Notch-transgenic mouse models to identify Notch targets in the BM CLPs and help uncover signaling pathways important in prethymic lymphoid progenitors.

We found that IL21r, expression of which we show to be Notch dependent both in vivo and in vitro, is a unique BM Notch target gene. IL21r signaling is involved in mature lymphoid cell processes, including T cell proliferation and CD8^+^ memory cell formation, NK cell maturation, macrophage phagocytosis, and context-dependent B cell proliferation, apoptosis, or plasma cell differentiation (57). IL-21 is a key component in immune responses to parasitic and chronic viral infection (95, 96). Although IL21r-KO mice have no defect in thymopoiesis, IL-21 signaling has been shown to affect CD4 and CD8 single-positive cell emigration (62, 97). Until our current study, a role for IL-21 signaling in BM CLPs had not been reported to our knowledge. We determined that IL21r expression was activated by Notch signaling specifically in the BM niche and that IL-21 treatment induced CLP proliferation in a Notch-dependent manner. We also confirmed that IL-21–induced expansion of CLP cells did not occur under homeostatic conditions in vivo. The lack of expression of IL-21 in T cells of neonatal pups makes it unlikely that this signaling axis plays a role in the early stages of postnatal developmental lymphopoiesis, although activation of the axis can be forced on CD34^+^ CB cells ex vivo through application of Notch ligands and recombinant IL-21. Additionally, the Notch/IL21r axis might not play a role in thymic progenitor culture of thymocytes using thymic epithelial cells ex vivo, due to the specificity of the signaling axis to BM progenitors (98). Given that BM-resident CD4^+^ TRM cells produce IL-21, it is likely that the context in which CLP proliferation downstream of IL21r signaling occurs is in response to inflammation and regenerative hematopoiesis. More work is required to confirm the context-dependent role for IL-21 signaling in CLP proliferation. Since IL-21–dependent expansion of BM CLP progenitors prior to thymic migration had not been previously reported to our knowledge, we set out to determine the extent to which addition of IL-21 ex vivo can be repurposed for expansion of functional mouse and human T cell progenitors.

Although injection of IL-21 in mice after HSCT has been shown to improve thymic T cell recovery in vivo, clinical application of such an approach may be untenable, as the pleiotropic mechanism of IL-21 signaling was shown not to be lineage specific, causing proliferation of BM LSK cells (99). Additionally, IL-21 injection in vivo could have off-target effects on mature lymphoid populations or even proliferative or survival effects in residual leukemic populations, such as in Waldenstrom macroglobulinemia, multiple myeloma, Hodgkin’s lymphoma, anaplastic large cell lymphoma, and adult T cell leukemia (100–106). Finally, IL-21 signaling can promote gastrointestinal GVHD in allogeneic transplants (107, 108). The ability to target specific cell populations with IL-21 activation, such as ex vivo–cultured progenitors, would be clinically advantageous and would avoid potential off-target IL21r activation in HSCT patients.

In summary, we discovered a new BM-specific Notch/IL-21 signaling axis and showed that IL-21 treatment provides a significant and dramatic improvement in the ex vivo expansion of functional T cell progenitors. As methods for ex vivo generation of T cell precursors require rare CB or mobilized BM-derived HSCs for starting material, methods that improve the total output of thymic seeding cells are advantageous for clinical feasibility. While Notch signals provide the vital T cell differentiation cues to early hematopoietic progenitors in the BM niche, we show how a discovery in vivo can be directly repurposed to improve an ex vivo platform and thus utilize IL-21–induced expansion of thymic seeding precursors. Therefore, both mouse and human hematopoietic progenitors can be primed and expanded for rapid T cell development, while retaining functional potential to support immune recovery in allogeneic transplants.

## Methods

### Mice.

Further information can be found in Supplemental Methods. B6.SJL-*Ptprc^a^* *Pep3^b^*/BoyJ (or CD45.1/.2) mice, C57BL/6J (or CD45.2) mice, and LP/J mice were purchased from The Jackson Laboratory. Breeding pairs of B6.129-*Il21r^tm1Kopf^*/J (IL21r-KO) were purchased from The Jackson Laboratory. Breeding pairs of Notch^+/ΔTAD^ mice were generated as described previously (25), and B6.129-*Notch1^tm1Con^*/J (Notch^+/–^) mice were obtained from The Jackson Laboratory. Notch1 conditional floxed mice were generated by crossing Notch1^fl/fl^ (gift from Jan Kitajewski, University of Illinois at Chicago, Chicago, Illinois, USA) to Vav-Cre mice (54). All mice were backcrossed to C57BL/6J mice for more than 10 generations before being used in our study. Both male and female mice were used, and all mice used were 6–12 weeks of age. They received food and water ad libitum. Mice were housed at the University of Illinois at Chicago (UIC) Association for Assessment and Accreditation of Laboratory Animal Care International–certified animal facilities.

### Preparation of BM, thymus, lymph node, and spleen mononuclear cells.

Femurs, tibias, spleens, and thymuses were harvested from mice immediately after euthanasia with 3%–5% isoflurane and by cervical dislocation. BM cells were flushed into PBS containing 2% fetal bovine serum using a 22-gauge needle (BD Biosciences) and syringe. Single-cell suspensions from spleens, thymuses, and inguinal lymph nodes were prepared by mincing and gently passing cells through 70 μm cell strainers (CELLTREAT Scientific Products). ACK lysing buffer (Invitrogen) was used to remove red blood cells to isolate mononuclear cells.

### Cell culture.

OP9 and OP9-Dll1 (a gift from Juan Carlos Zuniga-Pflucker, University of Toronto, Toronto, Ontario, Canada; and Warren Pear, University of Pennsylvania, Philadelphia, Pennsylvania, USA, at different times), and U2OS (ATCC) cells were cultured in Full Media: DMEM (Corning) supplemented with 10% FBS (Hyclone), 1× GlutaMAX (Gibco), and 1× penicillin/streptomycin (Corning). For all cocultures, OP9 or OP9-Dll1 cells were plated at a concentration of 2.78 × 10^4^ cells/cm^2^. For T cell progenitor differentiation, LSK cells were sorted at 3.1 × 10^3^ cells/cm^2^ directly into wells plated with OP9 or OP9-Dll1 24 hours earlier in Full Media supplemented with 1 ng/μL IL-7, 5 ng/μL Flt3L, with or without 20 ng/μL IL-21 (Peprotech). Fresh cytokines were added every 48 hours. For cultures lasting longer than 1 week, hematopoietic cells were gently pipetted to avoid removing feeder cells and placed in new wells plated with fresh feeder cells 24 hours prior. For cultures using plate-bound ligand, plates were prepared by incubating with 10 μg/mL recombinant human Dll4 and 2.32 μg/mL recombinant human VCAM-1 (R&D Systems) diluted in chilled PBS overnight at 4°C. Plates were washed once with PBS before cells were added. LSK cells were sorted and added at a concentration of 3.1 × 10^3^ cells/cm^2^ in serum-free IMDM (Gibco) supplemented with 20% bovine serum albumin, insulin, and transferrin serum substitute (StemCell Technologies); 1× GlutaMAX (Gibco); 1 μg/mL low-density lipoproteins (Calbiochem); 1 ng/mL IL-7; 5 ng/mL Flt3L; 25 ng/mL stem cell factor (SCF); with or without 20 ng/mL IL-21 (Peprotech). Cells were moved onto freshly prepared ligand-coated plates in fresh supplemented media every 96 hours. For human pro–T cell differentiation, 8.3 × 10^3^ freshly thawed human CD34^+^ cord blood cells (Lonza) were added to 12-well plates that had been plated with OP9-Dll1 cells 24 hours prior, in DMEM supplemented with 20% FBS, 1× GlutaMAX, 1× penicillin/streptomycin, 30 ng/mL human SCF, 10 ng/mL human IL-7, 10 ng/mL human Flt3L, with or without 20 ng/mL human IL-21 (Peprotech). Cells were placed in new wells plated with fresh feeder cells 24 hours prior in fresh supplemented media every 96 hours. For all ex vivo cultures using sorted mouse cells, 3–5 individual mice were used as biological replicates. For human CD34^+^ cord blood cultures, data were generated using cells collected from 2 individual donors.

### Ex vivo–derived T cell progenitor transplantation.

Indicated numbers of ex vivo–derived T cell progenitors (CD45.1/.2 or CD45.1) from control or IL-21–treated OP9-Dll1 cocultures were retro-orbitally transplanted into 4.5 Gy irradiated congenic recipients (CD45.1). For the allogeneic transplant, indicated numbers of ex vivo–derived T cell progenitors (CD45.1) and sorted BM HSCs (CD45.1/.2) were retro-orbitally transplanted into 9.0 Gy irradiated LP/J recipients (Ly9.1). All cells were washed with cold PBS prior to injection. Recipient mice were maintained on antibiotic water for 1 week before transplantation and 2 weeks posttransplant. To analyze engraftment ability of transplanted hematopoietic cells, peripheral lymphoid organs were collected at indicated time points after transplantation, and donor-derived T cell populations were analyzed by flow cytometry.

### RNA-Seq.

A total of 4 × 10^3^ to 11 × 10^3^ CLPs were sorted per mouse into RNA lysis buffer (Invitrogen) and stored at –80°C until RNA extraction. Total RNA was extracted using PicoPure RNA Isolation Kit (Applied Biosystems) with inclusion of RNase-free DNase (QIAGEN) treatment, as per manufacturer’s instructions. RNA samples were quantified using Quantus fluorometer (Promega). RNA-Seq was performed by the UIC at Chicago Genome Research Core. Sequencing libraries were prepared using strand-specific QuantSeq 3′ RNAseq kit (Lexogen, FWD, catalog 015). Total RNA in the amount of 1 nanogram per sample was used as an input. Library construction was performed according to the manufacturer’s protocol with all modifications recommended for samples with low RNA input.

In brief, during the first strand cDNA synthesis, an oligo dT primer containing an Illumina-compatible sequence at its 5′ end was hybridized to mRNA and reverse transcription was performed. After that, the RNA template was degraded, and during second strand synthesis, the library was converted to dsDNA. Second strand synthesis was initiated by a random primer containing an Illumina-compatible linker sequence at its 5′ end. The dsDNA libraries were purified by using magnetic beads to remove all reaction components. Next, the libraries were amplified to add the complete adapter sequences required for cluster generation and to generate sufficient material for quality control and sequencing. The number of PCR amplification cycles was 22, as determined by test quantitative PCR using a small preamplification library aliquot for each individual sample. Final amplified libraries were purified using purification beads supplied with the kit and quantified, and fragment sizes were confirmed to be within 263–318 bp by gel electrophoresis using Agilent 4200 TapeStation (D1000 Screen Tape). Concentration of the final library pool was confirmed by quantitative PCR. Sequencing was performed on NextSeq 500 (Illumina), high-output kit, 1 × 75 nt single reads.

RNA-Seq data were analyzed using the following tools from the BaseSpace Sequence Hub (Illumina): FASTQ adapter and quality trimming were performed using FASTQ Toolkit (V2.2.0), STAR alignment was performed using RNA-Seq Alignment (V2.0.0) using Mus_musculus (UCSC mm10) reference genome, and differential expression was performed using RNA-Seq Differential Expression (V1.0.1). Data were deposited in National Center for Biotechnology Information’s Gene Expression Omnibus GSE189370.

### Western blot analysis.

Whole-cell lysates were prepared with RIPA buffer with protease inhibitors. Protein concentration was determined with the Bio-Rad protein assay dye reagent (Bio-Rad). Proteins were separated using SDS-PAGE and transferred to PVDF membranes. Antibodies used for Western blot were cleaved Notch1 (Val1744) antibody (Cell Signaling Technology catalog 2421), GAPDH (MilliporeSigma catalog AB2302), and secondary anti-mouse–HRP (Bio-Rad catalog 170-6516) or anti-rabbit–HRP (Bio-Rad catalog 170-6515). Blots were visualized with SuperSignal west pico chemiluminescence (Thermo Fisher Scientific) or SuperSignal west femto chemiluminescence substrate (Thermo Fisher Scientific).

### qRT-PCR.

BM CLP, CD3^+^CD4^+^, CD3^+^CD8^+^, CD11b^+^, CD19^+^, and NK1.1^+^ cells, as well as thymic DN1s, DN2s, DN3s, and CD8^+^ cells, were sorted for RNA isolation using RNeasy Mini or Micro Kit (QIAGEN), except for BM CLP RNA, which was isolated with the PicoPure RNA kit (Thermo Fisher Scientific). BM CD3^+^ cells from P3 pups were bead purified, and RNA was isolated with the PicoPure RNA kit. RNA from cultured cells was isolated using RNeasy Mini Kit. cDNA was synthesized from RNA with the Super Script III kit (Invitrogen). Transcripts were amplified with SYBR Green qRT-PCR master mix (Applied Biosystems), and qRT-PCR was performed on the Viia7 system (Applied Biosystems). GAPDH or EF1α were used as housekeeping genes. Primer sequences are provided in Supplemental Table 2.

### Statistics.

The data were analyzed by ANOVA. Differences among group means were analyzed by Student-Newman-Keuls multiple comparisons test after 1- or 2-way ANOVA. For experiments in which only single experimental and control groups were used, group differences were examined by unpaired Student’s 2-tailed *t* test. Differences were considered significant at *P* < 0.05. All data represent mean ± SEM. All analyses were done with GraphPad Prism from GraphPad Software. Figures were created using GraphPad Prism and with BioRender.com.

### Study approval.

The Institutional Animal Care and Use Committee of UIC approved all animal procedures used in this study. Umbilical cord blood cells were obtained from Lonza. All cryopreserved umbilical cord blood samples were obtained from healthy, consenting donors and an IRB-approved source (Lonza 2C101B Batch 19TL190053).

## Author contributions

KS contributed to experimental design, generation of results, data analysis, and writing of the manuscript. NYP, TB, ZW, and LS contributed to generation of results. TS contributed to experimental design of allogeneic transplant and manuscript review. KVP contributed to experimental design and writing of the manuscript.

## Supplementary Material

Supplemental data

## Version 1 03/29/2022

In-Press Preview

## Version 2 05/09/2022

Electronic publication

## References

[1] Themeli M (2015). New cell sources for T cell engineering and adoptive immunotherapy. Cell Stem Cell.

[2] Zakrzewski JL (2008). Tumor immunotherapy across MHC barriers using allogeneic T-cell precursors. Nat Biotechnol.

[3] Shukla S (2017). Progenitor T-cell differentiation from hematopoietic stem cells using Delta-like-4 and VCAM-1. Nat Methods.

[4] Smith M (2018). Posttransplant chimeric antigen receptor therapy. Blood.

[5] Menon T (2015). Lymphoid regeneration from gene-corrected SCID-X1 subject-derived iPSCs. Cell Stem Cell.

[6] Iriguchi S (2021). A clinically applicable and scalable method to regenerate T-cells from iPSCs for off-the-shelf T-cell immunotherapy. Nat Commun.

[7] Reimann C (2012). Human T-lymphoid progenitors generated in a feeder-cell-free Delta-like-4 culture system promote T-cell reconstitution in NOD/SCID/γc(-/-) mice. Stem Cells.

[8] Dai X (2019). Standardizing CAR-T therapy: getting it scaled up. Biotechnol Adv.

[9] Sambandam A (2005). Notch signaling controls the generation and differentiation of early T lineage progenitors. Nat Immunol.

[10] Tan JB (2005). Requirement for Notch1 signals at sequential early stages of intrathymic T cell development. Nat Immunol.

[11] Radtke F (1999). Deficient T cell fate specification in mice with an induced inactivation of Notch1. Immunity.

[12] Kumar BV (2018). Human T cell development, localization, and function throughout life. Immunity.

[13] Rothenberg EV (2011). T cell lineage commitment: identity and renunciation. J Immunol.

[14] Yui MA (2010). Fine-scale staging of T cell lineage commitment in adult mouse thymus. J Immunol.

[15] Yui MA, Rothenberg EV (2014). Developmental gene networks: a triathlon on the course to T cell identity. Nat Rev Immunol.

[16] Amsen D (2015). Notch in T cell differentiation: all things considered. Trends Immunol.

[17] Hosokawa H, Rothenberg EV (2018). Cytokines, transcription factors, and the initiation of T-cell development. Cold Spring Harb Perspect Biol.

[18] Hozumi K (2008). Delta-like 4 is indispensable in thymic environment specific for T cell development. J Exp Med.

[19] Steinbuck MP, Winandy S (2018). A review of Notch processing with new insights into ligand-independent Notch signaling in T-cells. Front Immunol.

[20] Kopan R, Ilagan MXG (2009). The canonical Notch signaling pathway: unfolding the activation mechanism. Cell.

[21] Bray SJ (2006). Notch signalling: a simple pathway becomes complex. Nat Rev Mol Cell Biol.

[22] Mercher T (2008). Notch signaling specifies megakaryocyte development from hematopoietic stem cells. Cell Stem Cell.

[23] Cichocki F (2011). Cutting edge: microRNA-181 promotes human NK cell development by regulating Notch signaling. J Immunol.

[24] Kumano K (2003). Notch1 but not Notch2 is essential for generating hematopoietic stem cells from endothelial cells. Immunity.

[25] Gerhardt DM (2014). The Notch1 transcriptional activation domain is required for development and reveals a novel role for Notch1 signaling in fetal hematopoietic stem cells. Genes Dev.

[26] Sottoriva K, Pajcini KV (2021). Notch signaling in the bone marrow lymphopoietic niche. Front Immunol.

[27] Taqvi S (2006). Biomaterial-based Notch signaling for the differentiation of hematopoietic stem cells into T cells. J Biomed Mater Res A.

[28] Awong G (2009). Characterization in vitro and engraftment potential in vivo of human progenitor T cells generated from hematopoietic stem cells. Blood.

[29] Yu VWC (2015). Specific bone cells produce DLL4 to generate thymus-seeding progenitors from bone marrow. J Exp Med.

[30] Koo BK (2005). Mind bomb 1 is essential for generating functional Notch ligands to activate Notch. Development.

[31] Shao L (2019). A Tie2-Notch1 signaling axis regulates regeneration of the endothelial bone marrow niche. Haematologica.

[32] Yao D (2011). Protein O-fucosyltransferase 1 (Pofut1) regulates lymphoid and myeloid homeostasis through modulation of Notch receptor ligand interactions. Blood.

[33] Chen ELY (2019). RBPJ-dependent Notch signaling initiates the T cell program in a subset of thymus-seeding progenitors. Nat Immunol.

[34] Van Epps HL (2006). Bringing order to early B cell chaos. J Exp Med.

[35] Fathman JW (2011). Identification of the earliest natural killer cell-committed progenitor in murine bone marrow. Blood.

[36] Miyazaki M (2008). Thymocyte proliferation induced by pre-T cell receptor signaling is maintained through polycomb gene product Bmi-1-mediated Cdkn2a repression. Immunity.

[37] Inlay MA (2009). Ly6d marks the earliest stage of B-cell specification and identifies the branchpoint between B-cell and T-cell development. Genes Dev.

[38] Hu Y (2017). Deptor is a direct Notch1 target that promotes cell proliferation and survival in T-cell leukemia. Oncogene.

[39] Rochman Y (2009). New insights into the regulation of T cells by γc family cytokines. Nat Rev Immunol.

[40] Zhou L (2007). IL-6 programs TH-17 cell differentiation by promoting sequential engagement of the IL-21 and IL-23 pathways. Nat Immunol.

[41] Diehl SA (2008). STAT3-mediated up-regulation of BLIMP1 Is coordinated with BCL6 down-regulation to control human plasma cell differentiation. J Immunol.

[42] Nurieva RI (2008). Generation of T follicular helper cells is mediated by interleukin-21 but independent of T helper 1, 2, or 17 cell lineages. Immunity.

[43] Vogelzang A (2008). A fundamental role for interleukin-21 in the generation of T follicular helper cells. Immunity.

[44] Nurieva R (2007). Essential autocrine regulation by IL-21 in the generation of inflammatory T cells. Nature.

[45] Korn T (2007). IL-21 initiates an alternative pathway to induce proinflammatory T(H)17 cells. Nature.

[46] Fröhlich A (2009). IL-21R on T cells is critical for sustained functionality and control of chronic viral infection. Science.

[47] Kotlarz D (2013). Loss-of-function mutations in the IL-21 receptor gene cause a primary immunodeficiency syndrome. J Exp Med.

[48] Ryan RJH (2017). A B cell regulome links notch to downstream oncogenic pathways in small B cell lymphomas. Cell Rep.

[49] Messeguer X (2002). PROMO: detection of known transcription regulatory elements using species-tailored searches. Bioinformatics.

[50] Farré D (2003). Identification of patterns in biological sequences at the ALGGEN server: PROMO and MALGEN. Nucleic Acids Res.

[51] Rafei M (2013). Interleukin-21 accelerates thymic recovery from glucocorticoïd-induced atrophy. PLoS One.

[52] Holmes R, Zuñiga-Pflücker JC (2009). The OP9-DL1 system: generation of T-lymphocytes from embryonic or hematopoietic stem cells in vitro. Cold Spring Harb Protoc.

[53] Zeng R (2007). The molecular basis of IL-21-mediated proliferation. Blood.

[54] Ogilvy S (1999). Promoter elements of vav drive transgene expression in vivo throughout the hematopoietic compartment. Blood.

[55] Oh P (2013). In vivo mapping of notch pathway activity in normal and stress hematopoiesis. Cell Stem Cell.

[56] Varnum-Finney B (2011). Notch2 governs the rate of generation of mouse long- and short-term repopulating stem cells. J Clin Invest.

[57] Leonard WJ, Wan CK (2016). IL-21 signaling in immunity. F1000Res.

[58] Mueller SN, Mackay LK (2016). Tissue-resident memory T cells: local specialists in immune defence. Nat Rev Immunol.

[59] Steinbach K (2018). Resident-memory T cells in tissue-restricted immune responses: for better or worse?. Front Immunol.

[60] Mackay LK (2015). Cutting edge: CD69 interference with sphingosine-1-phosphate receptor function regulates peripheral T cell retention. J Immunol.

[61] Rettig MP (2012). Mobilization of hematopoietic stem and progenitor cells using inhibitors of CXCR4 and VLA-4. Leukemia.

[62] Ozaki K (2002). A critical role for IL-21 in regulating immunoglobulin production. Science.

[63] Zakrzewski JL (2006). Adoptive transfer of T-cell precursors enhances T-cell reconstitution after allogeneic hematopoietic stem cell transplantation. Nat Med.

[64] Zlotoff DA (2011). Delivery of progenitors to the thymus limits T-lineage reconstitution after bone marrow transplantation. Blood.

[65] Shale M (2013). CD4(+) T-cell subsets in intestinal inflammation. Immunol Rev.

[66] Maloy KJ, Powrie F (2011). Intestinal homeostasis and its breakdown in inflammatory bowel disease. Nature.

[67] Saleh M, Elson CO (2011). Experimental inflammatory bowel disease: insights into the host-microbiota dialog. Immunity.

[68] Weaver CT (2013). The Th17 pathway and inflammatory diseases of the intestines, lungs, and skin. Annu Rev Pathol.

[69] Izcue A (2009). Regulatory lymphocytes and intestinal inflammation. Annu Rev Immunol.

[70] Schmitt EG, Williams CB (2013). Generation and function of induced regulatory T cells. Front Immunol.

[71] Curotto de Lafaille MA, Lafaille JJ (2009). Natural and adaptive Foxp3+ regulatory T cells: more of the same or a division of labor?. Immunity.

[72] Sonderegger I (2008). IL-21 and IL-21R are not required for development of Th17 cells and autoimmunity in vivo. Eur J Immunol.

[73] Pott J, Stockinger S (2017). Type I and III interferon in the gut: tight balance between host protection and immunopathology. Front Immunol.

[74] Nava P (2010). Interferon-gamma regulates intestinal epithelial homeostasis through converging beta-catenin signaling pathways. Immunity.

[75] Farin HF (2014). Paneth cell extrusion and release of antimicrobial products is directly controlled by immune cell-derived IFN-γ. J Exp Med.

[76] Schulz SM (2008). IL-17A is produced by T_h_17, γδ T cells and other CD4^-^ lymphocytes during infection with *Salmonella enterica* serovar Enteritidis and has a mild effect in bacterial clearance. Int Immunol.

[77] Douzandeh-Mobarrez B, Kariminik A (2019). Gut microbiota and IL-17A: physiological and pathological responses. Probiotics Antimicrob Proteins.

[78] Sano T (2015). An IL-23R/IL-22 circuit regulates epithelial serum amyloid A to promote local effector Th17 responses. Cell.

[79] De Smedt M (2011). T-lymphoid differentiation potential measured in vitro is higher in CD34+CD38-/lo hematopoietic stem cells from umbilical cord blood than from bone marrow and is an intrinsic property of the cells. Haematologica.

[80] Thomas ED (1957). Intravenous infusion of bone marrow in patients receiving radiation and chemotherapy. N Engl J Med.

[81] Storek J (2008). Reconstitution of the immune system after hematopoietic stem cell transplantation in humans. Semin Immunopathol.

[82] Chaudhry MS (2017). Immune reconstitution after allogeneic hematopoietic stem cell transplantation: time to T up the thymus. J Immunol.

[83] Wilke C (2016). Marrow damage and hematopoietic recovery following allogeneic bone marrow transplantation for acute leukemias: effect of radiation dose and conditioning regimen. Radiother Oncol.

[84] Krenger W (2011). Thymic T-cell development in allogeneic stem cell transplantation. Blood.

[85] Storek J (1997). Infectious morbidity in long-term survivors of allogeneic marrow transplantation is associated with low CD4 T cell counts. Am J Hematol.

[86] Crawley AM (2014). Jak/STAT and PI3K signaling pathways have both common and distinct roles in IL-7-mediated activities in human CD8+ T cells. J Leukoc Biol.

[87] Alpdogan O (2001). Administration of interleukin-7 after allogeneic bone marrow transplantation improves immune reconstitution without aggravating graft-versus-host disease. Blood.

[88] Min D (2002). Protection from thymic epithelial cell injury by keratinocyte growth factor: a new approach to improve thymic and peripheral T-cell reconstitution after bone marrow transplantation. Blood.

[89] Dudakov JA (2015). Interleukin-22: immunobiology and pathology. Annu Rev Immunol.

[90] Zhang SL (2014). Chemokine treatment rescues profound T-lineage progenitor homing defect after bone marrow transplant conditioning in mice. Blood.

[91] Mackall CL (1997). Distinctions between CD8+ and CD4+ T-cell regenerative pathways result in prolonged T-cell subset imbalance after intensive chemotherapy. Blood.

[92] Awong G (2013). Human proT-cells generated in vitro facilitate hematopoietic stem cell-derived T-lymphopoiesis in vivo and restore thymic architecture. Blood.

[93] Simons L (2019). Concise review: boosting T-cell reconstitution following allogeneic transplantation—current concepts and future perspectives. Stem Cells Transl Med.

[94] Wang H (2006). Distinct roles of IL-7 and stem cell factor in the OP9-DL1 T-cell differentiation culture system. Exp Hematol.

[95] Elsaesser H (2009). IL-21 is required to control chronic viral infection. Science.

[96] Solaymani-Mohammadi S (2019). Interleukin (IL)-21 in inflammation and immunity during parasitic diseases. Front Cell Infect Microbiol.

[97] Moretto MM (2019). Complex and multilayered role of IL-21 signaling during thymic development. J Immunol.

[98] Villegas JA (2018). Cultured human thymic-derived cells display medullary thymic epithelial cell phenotype and functionality. Front Immunol.

[99] Tormo A (2017). Interleukin-21 promotes thymopoiesis recovery following hematopoietic stem cell transplantation. J Hematol Oncol.

[100] Ueda M (2005). Expression of functional interleukin-21 receptor on adult T-cell leukaemia cells. Br J Haematol.

[101] Bard JD (2009). IL-21 contributes to JAK3/STAT3 activation and promotes cell growth in ALK-positive anaplastic large cell lymphoma. Am J Pathol.

[102] Scheeren FA (2008). IL-21 is expressed in Hodgkin lymphoma and activates STAT5: evidence that activated STAT5 is required for Hodgkin lymphomagenesis. Blood.

[103] Ménoret E (2008). IL-21 stimulates human myeloma cell growth through an autocrine IGF-1 loop. J Immunol.

[104] Brenne AT (2002). Interleukin-21 is a growth and survival factor for human myeloma cells. Blood.

[105] Hodge LS (2012). IL-21 in the bone marrow microenvironment contributes to IgM secretion and proliferation of malignant cells in Waldenstrom macroglobulinemia. Blood.

[106] Croce M (2015). IL-21: a pleiotropic cytokine with potential applications in oncology. J Immunol Res.

[107] Hippen KL (2012). Blocking IL-21 signaling ameliorates xenogeneic GVHD induced by human lymphocytes. Blood.

[108] Hanash AM (2011). Abrogation of donor T-cell IL-21 signaling leads to tissue-specific modulation of immunity and separation of GVHD from GVL. Blood.

