# Supplementary material for: A Notch/IL-21 signaling axis primes bone marrow T cell progenitor expansion
Source: JCI Insight. 2022 May 9;7(9):e157015. doi: 10.1172/jci.insight.157015 (PMC9090257; doi:10.1172/jci.insight.157015)
Supplement: Supplemental data [file jciinsight-7-157015-s039.pdf]

Supplementary Methods

Irradiation

Mice were exposed to a total body irradiation (TBI) sub-lethal (4.5 Gy) or lethal (9.0 Gy) dose in a Mark I 137Ce γ-irradiator (JL Shepherd, Glendale, CA, USA) at a rate of 6.38 Gy/min. Mice were irradiated on a rotating platform.

Isolation of Lamina Propria (LP) Mononuclear cells from intestinal tissue

Lamina Propria mononuclear cells were isolated from the ileum of small intestine (SI) and large intestine (LI) including cecum following the procedure described in previous study (1). Briefly, all the chains of MLNs were first collected from the mice and the SI was separated from LI. SI was further divided into three equal parts: Duodenum (first 1/3 connecting to stomach), Jejunum (middle 1/3), and Ileum (last 1/3 connecting to cecum). Attaching fat tissues were removed from the intestine. Tissues were cut into 3-4 pieces (5-8 cm), placed in PBS containing 1mM DTT and 5mM EDTA (No magnesium and calcium), and incubated at 37°C on a shaker with vigorous shaking (225-235 rpm) for 20 min. This process was repeated once with vigorous shaking (225-235 rpm) for 10 min. Subsequently, tissues were washed in RPMI supplemented with 2% FBS, to remove DTT and EDTA. The incubated tissues were digested in CM10: RPMI supplemented with 10% FBS,1xMEM, 10 mM HEPES, 1 mM Sodium Pyruvate, 1000 U/mL Pencillin and 1000mg/mL Streptomycin) with 1mg/mL Collagenase D, 100ug/mL DNase, and 0.01 U/mL Dispase at 37°C on a shaker (225-235 rpm) for 30 minutes for the ileum and 45 minutes for LI. After digestion, the digestive reaction was stopped/delayed by adding 14 mL of ice cold RPMI 10% FBS. The tissues were then passed through a 70 µM cell strainer and mononuclear lymphocytes were isolated using a Percoll density gradient of 40%:80%. After aspiration of the unnecessary cells and tissues stacked in the top of 40% Percoll, the Lymphocytes were collected from intermediate layer, washed with ice cold RPMI 10% FBS, and counted using Trypan Blue and the Countess II Automated Cell Counter.

Isolation of Mononuclear cells from Mesenteric Lymph Nodes (MLN)

MLNs were extracted from SI before it was processed and stored on ice in RPMI 10% FBS. MLNs were dissociated by using the plunger from a 3 mL syringe and pressing the tissues through a 70 µM cell strainer, washing with 5mL CM10. MLN cells were then subjected to a Percoll density gradient of 40%:80% to isolate mononuclear lymphocytes. Following collection of the lymphocyte interphase, it was then washed again in CM10 and counted using Trypan

blue and the Countess II Automated Cell Counter.

Isolation of CD25+ thymocytes and CD3+ BM cells from P3 pups

Cell suspensions from thymus or P3 crushed long bones were incubated with CD25 antibody- and CD3 antibody-labelled dynabeads (Invitrogen) for 30 min at 4°C, respectively. CD25+ thymocytes were isolated and used for western blot and P3 BM CD3+ cells were isolated and used for qRT-PCR.

Flow cytometry

Bone marrow: mononuclear cells were isolated as described above. Cells were preincubated with anti-CD16/32 antibody to block the Fcγ receptors. They were then incubated with anti-CD3e, anti-CD45R/B220, anti-Gr-1, anti-CD11b, and anti-Ter119 antibodies, and stained with appropriate antibody combinations to analyze HSC (Lin-/Sca1+/cKit+/CD150+/CD48-), LSK (Lin-/Sca1+/cKit+), LMPP (Lin-/Sca1+/cKit+/CD135+), CLP (Lin-/Sca1Lo/ckitLo/CD127+/CD135+), Hardy Fraction A (B220+/CD43+/BP-1-/CD24-), Hardy Fraction B (B220+/CD43+/BP-1-/CD24+), pre-NKP (Lin-/CD244+/CD27+/CD135-/CD122-), rNKP (Lin-/CD244+/CD27+/CD135-/CD122+), and CD4+ TRM (CD3+/CD4+/CD44+/CD69+/CD62L-/CD49d+/CD29+) for 30 minutes at 4°C. For surface receptor staining, cells were additionally stained for anti-IL21r, anti-Notch1, or anti-Notch2 for 30 minutes at 4°C. For intracellular staining, cells were first stained for live/dead and surface markers, and then fixed in FluoroFix Buffer (Biolegend), permeabilized in Intracellular Staining Permeabilization Wash (Biolegend), and then stained with anti-Ki67 or anti-IL21 according to manufacturer's instructions. For intracellular cleaved ICN1 staining, cells were stained, fixed, and permeabilized as described above, and then incubated with Cleaved Notch1 (Val1744, Cell Signaling Technologies) for 30 minutes at 4°C, washed, and then incubated with secondary anti-Rabbit for 30 minutes at 4°C. For intracellular IL21 staining, BM cells were first cultured for 5 Hrs in Cell Activation Cocktail with Brefeldin A (Biolegned) containing media before subsequent surface staining and fixation/permeabilization. Flow data was collected on a Fortessa LSRII analyzer (BD Biosciences), and the frequencies of cell populations were analyzed by FlowJo (FlowJo, Ashland, OR) software. Absolute cell numbers were calculated by multiplying CLP percentage of live cells by of the total numbers of bone marrow cells from each mouse. ZombieAqua (Biolegend) was used to exclude dead cells according to manufacturer's instructions. Detailed antibody information is provided in the Supplemental Table 1.

Thymus: Thymocyte suspensions were made as described above. Thymocytes were

preincubated with anti-CD16/32 antibody to block the Fcγ receptors and then incubated with anti-CD45.1, anti-CD45.2, anti-Ly9.1, anti-CD45R/B220, anti-Gr-1, anti-CD11b, and anti-Ter119 antibodies, and stained with appropriate antibody combinations to analyze DP (Lin-/CD4+/CD8+), DN (Lin-/CD4-/CD8-), and DN3 cells (Lin-/CD4-/CD8-/CD25+/CD44-) for 30 minutes at 4°C. For surface receptor staining, cells were additionally stained for anti-IL21r, anti-Notch1, or anti-Notch2 for 30 minutes at 4°C. Frequencies of DN3 cells were analyzed with a Fortessa LSRII analyzer and FlowJo software. Absolute cell numbers of each population from each mouse were calculated by percentages of each population multiply total numbers of thymocytes from each mouse. Fortessa LSRII analyzer and FlowJo software were used to analyze surface expression of IL21r, Notch1, and Notch2 on DN3 cells. DAPI was used to exclude dead cells. Detailed antibody information is provided in the Supplemental Table 1.

Spleen: Splenocyte suspensions were made as described above. Splenocytes were preincubated with anti-CD16/32 antibody to block the Fcγ receptors and then incubated with anti-CD45.1, anti-CD45.2, anti-Ly9.1, anti-CD3, anti-CD4, and anti-CD8 antibodies for 30 minutes at 4°C. Frequencies of T cells were analyzed with a Fortessa LSRII analyzer and FlowJo software. Absolute cell numbers of each population from each mouse were calculated by percentages of each population multiplied by total numbers of splenocytes from each mouse. DAPI was used to exclude dead cells. Detailed antibody information is provided in the Supplemental Table 1.

Lymph nodes: Inguinal lymph node suspensions were made as described above. Lymph node cells were preincubated with anti-CD16/32 antibody to block the Fcγ receptors and then incubated with anti-CD45.1, anti-CD45.2, and anti-CD3 antibodies for 30 minutes at 4°C. Frequencies of T cells were analyzed with a Fortessa LSRII analyzer and FlowJo software. Absolute cell numbers of each population from each mouse were calculated by percentages of each population multiply total numbers of cells in inguinal lymph nodes from each mouse. DAPI was used to exclude dead cells. Detailed antibody information is provided in the Supplemental Table 1.

Peripheral blood: Peripheral blood was collected via tail vein and red blood cells were removed by ACK lysis buffer. Cells were preincubated with anti-CD16/32 antibody to block the Fcγ receptors and then stained with anti-CD45R/B220, anti-CD4, anti-CD8, anti-CD3, and anti-NK1.1 antibodies for 30 minutes at 4°C. Data analysis was performed using a Fortessa LSRII analyzer and FlowJo software. DAPI was used to exclude dead cells. Detailed antibody information is provided in the Supplemental Table 1.

Cultured cells: Suspensions of hematopoietic cells were made by gently pipetting suspended cells and passing through 70 μm cell strainers. Plates were then washed twice with PBS, which was passed through the same cell strainer. Cells were stained with anti-CD44, anti-CD25, anti-CD11b, and anti-B220. For intracellular staining, cells were first stained for ZombieAqua (Biolegend) and surface markers, and then fixed in FluoroFix Buffer (Biolegend), permeabilized in Intracellular Staining Permeabilization Wash (Biolegend), and then stained with anti-pThr202/Tyr204 ERK, anti-pSer727 STAT1 and anti-pTyr705 STAT3 according to manufacturer's instructions. Data analysis was performed using a Fortessa LSRII analyzer and FlowJo software. Absolute cell numbers were calculated by percentages of each population multiplied by total numbers of cells per well. DAPI was used to exclude dead cells. Detailed antibody information is provided in the Supplemental Table 1.

Detection of cytokines produced in T cells

After isolation of mononuclear cells from SI, LI, and MLN, $1x10^6$ mononuclear cells per sample were plated on a 96 well round-bottom plate. The cells were restimulated for 4 hours at 37°C with CM10 supplemented with PMA (50ng/mL), Ionomycin (500ng/mL), and GolgiPlug (1x). Following restimulation, cells were washed with CM10 then incubated with anti-CD16/32 antibody to block the Fcγ receptors and Goat Anti-Armenian Hamster IgG for 15 minutes at 4°C. The cells were washed with CM10 and extracellular markers were stained using anti-CD45.2, anti-TCRβ, anti-Ly9.1, anti-MHC II, anti-CD4, and anti-CD45.1 antibodies for 30 minutes at 4°C. For intracellular staining, cells were first stained for live/dead and surface markers and fixed with BD Fixation Kit for 30 minutes in the dark at 4°C. The cells were then permeabilized using BD fixation Kit Permeabilization Buffer. Finally, the cells were stained for intracellular markers with anti-IFN-γ and anti-IL-17A according to manufacturer's instructions. The cells were washed using BD permeabilization buffer and filtered with 70μM cell strainer before running on Attune NxT Cytometer (Invitrogen). Absolute cell numbers of each population were calculated by percentages of each population multiplied by total numbers of cells in each corresponding tissue from each mouse. Aqua Live/Dead stain (Invitrogen) was used to exclude dead cells according to manufacturer's instructions. Detailed antibody information is provided in the Supplemental Table 1.

Detection of cell surface markers and transcription factors in T cells

After isolation of mononuclear cells from SI, LI, and MLN, $1x10^6$ mononuclear cells per sample were plated on a 96 well round-bottom plate. The cells were immediately incubated with anti-

CD16/32 antibody to block the Fcγ receptors and Goat Anti-Armenian Hamster IgG for 15 minutes at 4°C. The cells were washed with CM10 and extracellular markers were stained using anti-CD44, anti-TCRβ, anti-CD45.2, anti-MHC II, anti-CD4, anti-Ly9.1, and anti-CD45.1 antibodies for 30 minutes in the dark at 4°C. For intracellular staining, cells were first stained for live/dead and surface markers and fixed with eBioscience Fixation Kit for 30 minutes in the dark at room temperature. The cells were permeabilized with eBioscience Permeabilization Buffer. Finally, the cells were stained for intracellular markers with anti-GATA3, anti-FOXP3, anti-RORγt, and anti-Tbet according to manufacturer's instructions. The cells were washed using eBioscience permeabilization buffer and filtered with 70μM cell strainer before running on Attune NxT Cytometer (Invitrogen). Absolute cell numbers of each population were calculated by percentages of each population multiplied by total numbers of cells in each corresponding tissue from each mouse. Aqua Live/Dead stain (Invitrogen) was used to exclude dead cells according to manufacturer's instructions. Detailed antibody information is provided in the Supplemental Table 1.

Luciferase assay

U2OS cells were seeded $1x10^4$ cells per well in 96-well plates, with 5-6 wells per condition. Four hours after seeding, the medium was changed, and cells were transfected with Fugene-6 with combinations of the following plasmids, as specified in Supplementary Figure 2C-D: pGL3-CSL4X, pGL3-IL21r-WT, pGL3-IL21r-Mut, pcDNA3-Empty Vector (EV), pcDNA3-ICN1, pcDNA3-ICN2, pcDNA3-ICN1ΔTAD, pcDNA3-dnMAML and internal transfection control pRL-TK. pGL3-IL21r was made by cloning a 470bp gBlock (IDT) corresponding to the RBPj site containing region of human IL21r into the pGL3 backbone. One day post-transfection, the medium was changed, and 48 h post-transfection, the cells were lysed, and firefly luciferase levels were measured in one step with Britelite plus (PerkinElmer). Renilla luciferase was measured with Stop-n-Glo reagent from Promega. All readings were made using a Promega Glomax 96-microplate Luminometer at 1-sec intervals.

Supplementary Tables

Table 1. List of antibodies used to stain various types of cells

Markers	Clone	Antibody isotype	Conjugate
CD45R/B220[1]	RA3-6B2	IgG_{2a}	PE

CD3e[1]	17A2	IgG_1	PE
CD3e[1]	17A2	IgG_1	APC-CY7
CD11b[1]	M1/70	IgG_{2b}	PE
CD11b[1]	M1/70	IgG_{2b}	PE-CY7
Gr-1[1]	RB6-8C5	IgG_{2b}	PE
Ter-119[1]	Ter-119	IgG_{2b}	PE
Ly6D[1]	49-H4	IgG_{2c}	PE
CD19[1]	6D5	IgG_{2a}	PE
CD19[1]	6D5	IgG_{2a}	PE-CY7
NK1.1[1]	PK136	IgG_{2a}	PE
CD45R/B220[1]	RA3-6B2	IgG_{2a}	FITC
CD45R/B220[1]	RA3-6B2	IgG_{2a}	APC
CD45R/B220[1]	RA3-6B2	IgG_{2a}	APC-CY7
CD3e[1]	17A2	IgG_1	FITC
CD11b[1]	M1/70	IgG_{2b}	FITC
Gr-1[1]	RB6-8C5	IgG_{2b}	FITC
Ter-119[1]	Ter-119	IgG_{2b}	FITC
CD16/CD32[1]	93	IgG_{2a}	purified
CD43[1]	S11	IgG_{2b}	APC
BP-1[1]	6C3	IgG_{2a}	PE-CY7
CD24[2]	30-F1	IgG_{2c}	PE

CD244[1]	m2B4 (B6)458.1	IgG_1	PE-CY7
CD27[1]	LG.3A10	IgG	PerCP-Cy5.5
CD122[1]	TM-β1	IgG_{2b}	FITC
CD45.2[1]	104	IgG_{2a}	FITC
CD45.2[1]	104	IgG_{2a}	Alexa Fluor 700
CD45.2[1]	104	IgG_{2a}	APC-CY7
CD45.2[1]	104	IgG_{2a}	Pacific Blue
CD45.1[1]	A20	IgG_{2a}	PerCP-Cy5.5
CD45.1[1]	A20	IgG_{2a}	PE/Dazzle 594
CD45.1[1]	A20	IgG_{2a}	APC
CD45.1[1]	A20	IgG_{2a}	PE-CY7
CD45.1[1]	A20	IgG_{2a}	PE
Ly9.1[3]	30C7	IgG_{2a}	BV421
Sca-1[1]	D7	IgG_{2a}	PerCP-Cy5.5
c-kit[1]	2B8	IgG_{2b}	APC-CY7
c-kit[1]	2B8	IgG_{2b}	FITC
c-kit[1]	2B8	IgG_{2b}	PE
CD135[1]	A2F10	IgG_{2a}	PE
CD135[1]	A2F10	IgG_{2a}	BV421
CD127[1]	A7R34	IgG_{2a}	PE-CY7
CD48[1]	HM48-1	IgG	Alexa Fluor 700

CD150[1]	TC15-12F12.2	IgG_{2a}	Alexa Fluor 647
CD150[1]	TC15-12F12.2	IgG_{2a}	BV605
CD4[1]	GK1.5	IgG_{2b}	PE-CY7
CD8a[1]	53-6.7	IgG_{2a}	APC-CY7
CD44[1]	IM7	IgG_{2b}	PerCP-Cy5.5
CD44[1]	IM7	IgG_{2b}	Alexa Fluor 700
CD25[1]	PC61	IgG_1	Alexa Fluor 700
CD25[1]	PC61	IgG_1	APC
CD69[1]	H1.2F3	IgG	Pacific Blue
CD62L[1]	MEL-14	IgG_{2a}	PE/Dazzle 594
CD49d[1]	R1-2	IgG_{2b}	PE
CD29[2]	HMb1-1	IgG	PerCP-eFluor 710
IL21[1]	FFA21	IgG_{2a}	APC
pERK[1]	6B8B69	IgG_{2a}	FITC
pSTAT1[1]	A15158B	IgG_1	Alexa Fluor 647
pSTAT3[1]	13A3-1	IgG_1	PE
Isotype[1]	HTK888	IgG	PE
Isotype[1]	MOPC-21	IgG_1	APC
CD71[1]	RI7217	IgG_{2a}	APC
Notch1[1]	HMN1-12	IgG	APC

Notch2[1]	HMN2-35	IgG	APC
Ki67[1]	16A8	IgG_{2a}	Alexa Fluor 700
CCR7[1]	4B12	IgG_{2a}	PE/Dazzle 594
CCR9[1]	9B1	IgG_{2a}	FITC
PSGL1[2]	4RA10	IgG_{1}	PE
Cleaved Notch1 (Val1744)[4]	D3B8	IgG	N/A
Anti-Rabbit IgG[1]	Poly4064	Ig	Alexa Fluor 647
Human CD5[1]	L17F12	IgG_{2a}	APC-CY7
Human CD7[1]	CD7-6B7	IgG_{2a}	PerCP-Cy5.5
Human CD34[1]	581	IgG_{1}	FITC
IFNγ[2]	XMG1.2	IgG_{1}	Alexa Fluor 488
IL-17a[1]	TC11-18H10.1	IgG_{1}	Alexa Fluor 700
TCRβ[2]	H57-597	IgG	APC-eFluor 780
MHCII[3]	M5/114.15.2	IgG_{2b}	BV510
CD4[3]	RM4-5	IgG_{2a}	BV605
GATA3[2]	TWAJ	IgG_{2b}	Alexa Fluor 488
CD44[2]	IM7	IgG_{2b}	PerCP-Cy5.5
FOXP3[2]	FJK-16s	IgG_{2a}	APC
TCRβ[3]	H57-597	IgG_{2}	Alexa Fluor 700
RORγt[3]	Q31-378	IgG_{2a}	BV421

Tbet[2]	4B10	IgG_1	PE

Footnotes: [1] Biolegend, San Diego, CA; [2]eBioscience, San Jose, CA; [3]BD Bioscience, Becton, NJ; [4]Cell Signaling Technology, Danvers, MA

Table 2. Sequences of the primers used for qRT-PCR (5'-3')

Genes	Forward sequences	Reverse sequences
Deptor	ACCCCAAATCTGTGCTGAAGA	AACTGCGTCACCAACAATCG
IL21r	CACCTGACTGAACTCCTGCC	TGCTGTGTCCCAGACCTACT
Hes1	GAAAGATAGCTCCCGGCATT	GTCACCTCGTTCATGCACTC
Notch1	CTGTCCTCTGCCATATACAGGAGC	ACCTCGCAGGTTTGACCTTGCCAG
CD3	GACTATGAGCCCATCCGCAA	AACAAGGAGTAGCAGGGTGC
IL21	AGCACATAGCTAAATGCCCTTC	CCCGGACACAACATGGAAGT
Bcl11b	CCAGTGTGAGTTGTCAGGTAAAG	TCGGAAGCCATGTGTGTTCT
Tcf7	TCAAGAATCCACCACAGGAGG	AGCCTAGAGCACTGTCATCG
E2A	GAGGCCTTTAAGGAGCTCGG	TTCAGGTTGCGTTCTCGCAC
Ef1α	CACTTGGTCGCTTTGCTGTT	CGTGGCAGGTGTTAGGGGTA
GAPDH	TAAACTCAGGAGAGTGTTTCCTCG	ACTGCAAATGGCAGCCCTGGTGAC

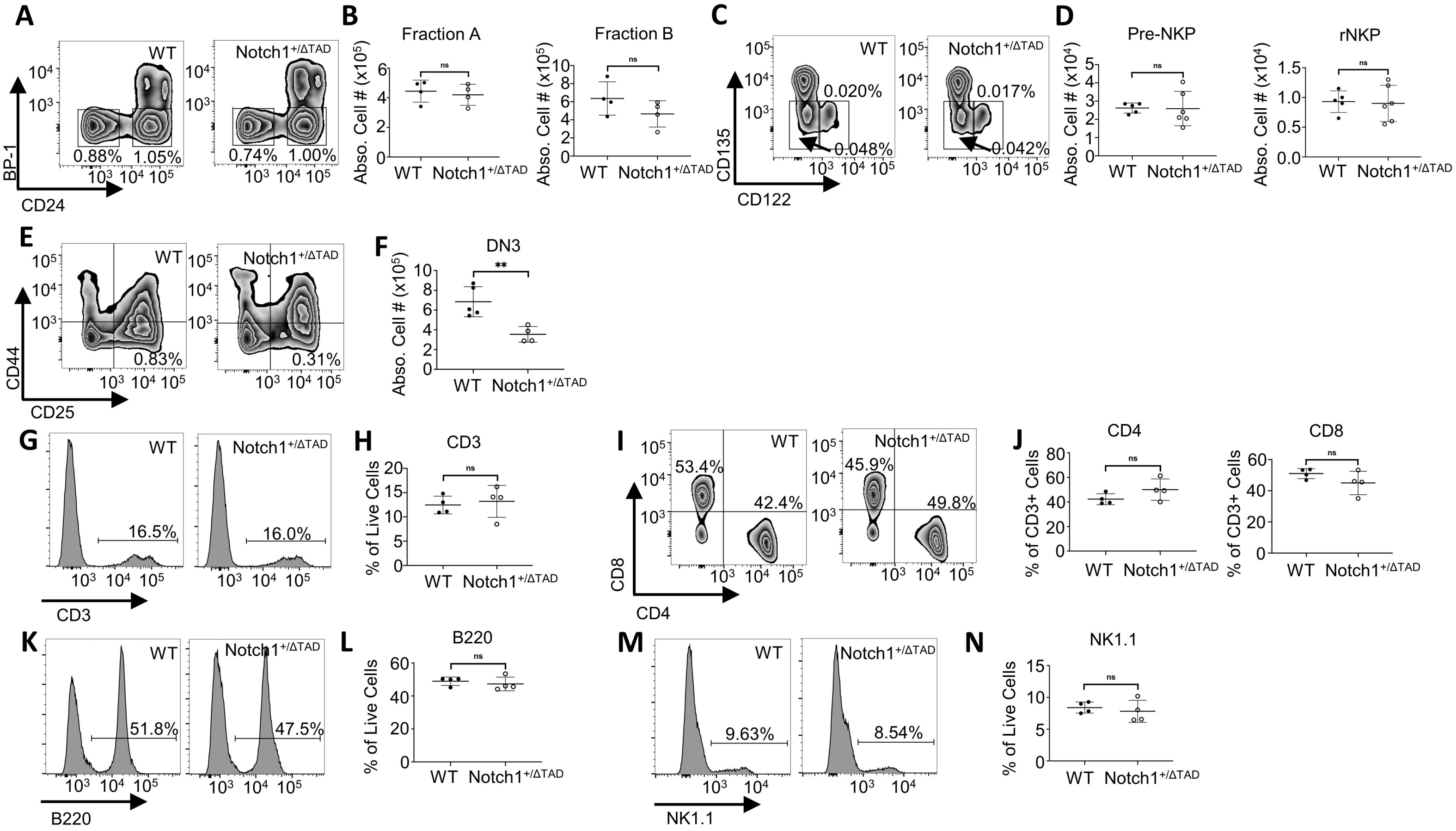

Supplemental Figure 1. Notch1$^{+/\Delta TAD}$ mice have thymic progenitor defect.

A. Representative flow plot for BM Fractions A (BP-1-/CD24-) and B (BP-1-/CD24+), gated on B220+/CD43+ with percentage of live cells shown. **B**. Absolute numbers of BM Fractions A and B in WT and Notch1$^{+/\Delta TAD}$ (n=4) mice. **C**. Representative flow plot for BM Pre-NKP (CD135-/CD122-) and rNKP (CD135-/CD122+), gated on Lin-/CD244+/CD27+ with percentage of live cells shown. **D**. Absolute numbers of BM Natural Killer progenitors in WT and Notch1$^{+/\Delta TAD}$ (n=4) mice. **E**. Representative plot of thymic DN3 population (CD44-/CD25+), gated on Lin-/CD4-/CD8- with percentage of live cells shown. **F**. Absolute number of thymic DN3 cells in WT (n=5) and Notch1$^{+/\Delta TAD}$ (n=4) mice. **G**. Representative histogram of CD3 staining of peripheral blood collected from WT or Notch1$^{+/\Delta TAD}$ mice, gated on live cells. **H**. Percentage of CD3+ cells in peripheral blood of WT or Notch1$^{+/\Delta TAD}$ (n=4). **I**. Representative flow plot of CD4 and CD8 staining of peripheral blood collected from WT or Notch1$^{+/\Delta TAD}$ mice, with percentage of CD3+ shown. **J**. Percentage of CD4+ (left) and CD8+ (right) cells in peripheral blood of WT or Notch1$^{+/\Delta TAD}$ (n=4). **K**. Representative histogram of B220 staining of peripheral blood collected from WT or Notch1$^{+/\Delta TAD}$ mice, gated on live cells. **L**. Percentage of B220+ cells in peripheral blood of WT or Notch1$^{+/\Delta TAD}$ (n=4). **M**. Representative histogram of NK1.1 staining of peripheral blood collected from WT or Notch1$^{+/\Delta TAD}$ mice, gated on live cells. **N**. Percentage of NK1.1+ cells in peripheral blood of WT or Notch1$^{+/\Delta TAD}$ (n=4). ns $p>0.05$, ** $p<0.01$. Statistical analysis performed using student t-test (**B-N**).

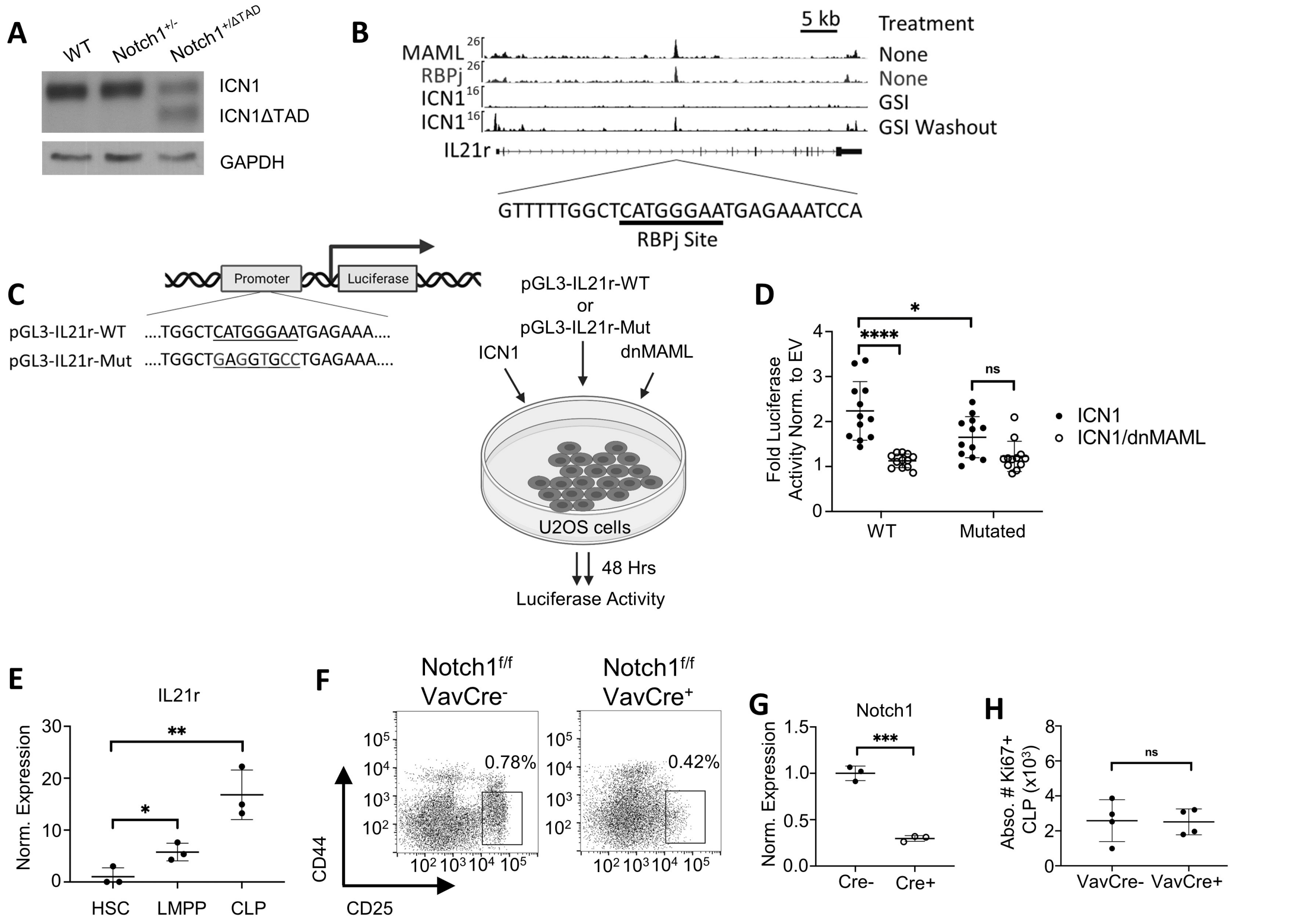

Supplemental Figure 2. Validation of IL21r RBPj binding site.

A. Western blot of protein from magnetic bead purified CD25+ thymus cells from WT, Notch1$^{+/-}$, and Notch1$^{+/\Delta TAD}$ mice. Top = cleaved ICN1, bottom = GAPDH **B**. RBPj, MAML, and ICN1 binding to the IL21r locus of Rec-1 cells treated with or without GSI, as determined by ChIP-seq. A single consensus RBPj site (underlined) was found within the peak region of binding of each protein to intron 2 of IL21r. **C**. U2OS cells were transfected with reporter pGL3-IL21r-WT or pGL3-IL21r-Mut, and with either empty control vector, ICN1 or both ICN1 and dnMAML. **D**. Firefly luciferase activity normalized to renilla activity and empty vector in U2OS cells transfected with indicated plasmids (n=12). **E**. IL21r expression relative to EF1α and normalized to HSC between sorted HSC, LMPP, and CLP cells from WT BM, determined by qRT-PCR (n=3) **F**. Representative plot of thymic DN3 population (CD44-/CD25+) in Notch1^{ff}VavCre^{-} or Notch1^{ff}VavCre^{+} mice, gated on Lin-/CD4-/CD8- with percentage of live cells shown. **G**. Notch1 expression relative to GAPDH and normalized to Notch1^{ff}VavCre^{-} between Notch1^{ff}VavCre^{-} or Notch1^{ff}VavCre^{+} sorted DN3 cells, determined by qRT-PCR (n=3). **H**. Absolute number of Ki67+ BM CLP cells in VavCre^{-} (n=4) and VavCre^{+} (n=4) mice. *** $p<0.001$. Statistical analysis performed using student t-test (**E,G-H**) and Two-way Anova (**D**).

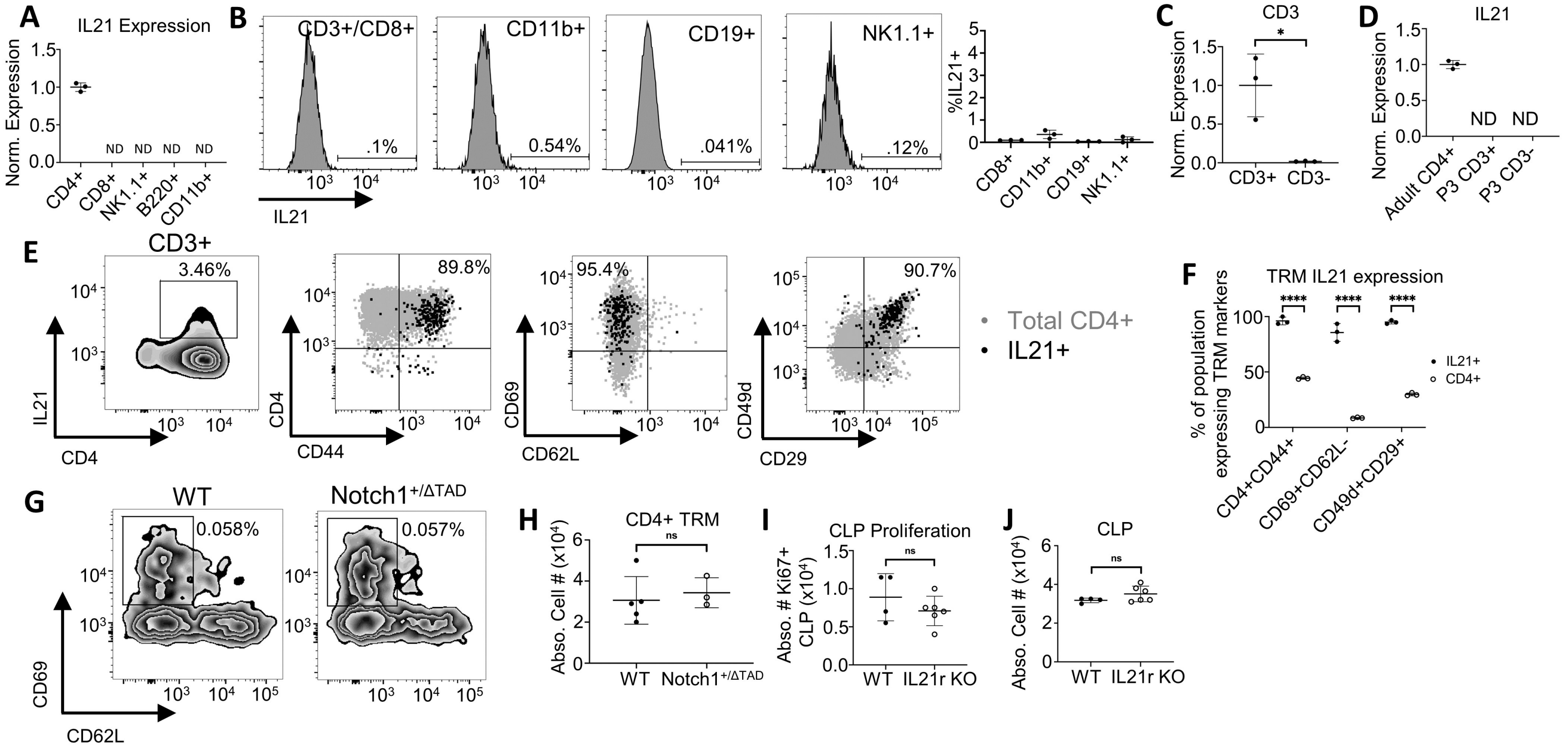

Supplemental Figure 3. Adult BM tissue resident memory CD4+ T cells produce IL21.

A. Expression of IL21 relative to GAPDH and normalized to CD4+ determined by qRT-PCR of mRNA from indicated BM populations sorted from WT (n=3) mice. **B**. (Left) Representative histograms for intracellular IL21 staining of selected populations from total BM cells. (Right) Percentage of indicated populations expressing intracellular IL21 (n=3). **C**. Expression of CD3 relative to GAPDH and normalized to CD3+ determined by qRT-PCR of mRNA from magnetically purified CD3+ or CD3- BM cells collected from P3 WT pups (n=3). **D**. Expression of IL21 relative to GAPDH and normalized to adult CD4+ determined by qRT-PCR of mRNA from magnetically purified CD3+ or unpurified BM cells collected from P3 WT pups and CD4+ cells sorted from WT adult BM (n=3) **E**. (left) Representative plot for intracellular IL21 of CD4 magnetically purified BM cells. Gated on CD3+. (right) Representative flow plots for TRM T cell markers (CD44+/CD69+/CD62L-/CD49d+/CD29+) of CD4 magnetically purified BM cells. Total intracellular IL21+ cells (black) are overlayed on total CD4+ cells (grey). Values indicate percentage of IL21+ cells in quadrant. **F**. Percentage of total CD4+ or total IL21+ cells expressing CD4+/CD44+, CD69+/CD62L-, or CD49d+/CD29+ (n=3). **G**. Representative flow plot for CD4+ TRM cells from WT or Notch1$^{+/\Delta TAD}$ BM cells. Gated on CD3+/CD4+/CD8-/CD44+/CD49d+/CD29+ with percentage of live cells shown. **H**. Absolute numbers of CD4+ TRM cells in BM of WT (n=5) or Notch1$^{+/\Delta TAD}$ (n=3) mice. **I**. Absolute number of Ki67+ BM CLP cells in WT (n=4) and IL21r KO (n=6) mice. **J**. Absolute number of total BM CLP cells in WT (n=4) and IL21r KO (n=6) mice. ND = Not Detected. TRM = Tissue Resident Memory. ns p>0.05, * p<0.05, **** p<0.0001. Statistical analysis performed using student t-test (**C,H-J**) and One-way Anova (**F**).

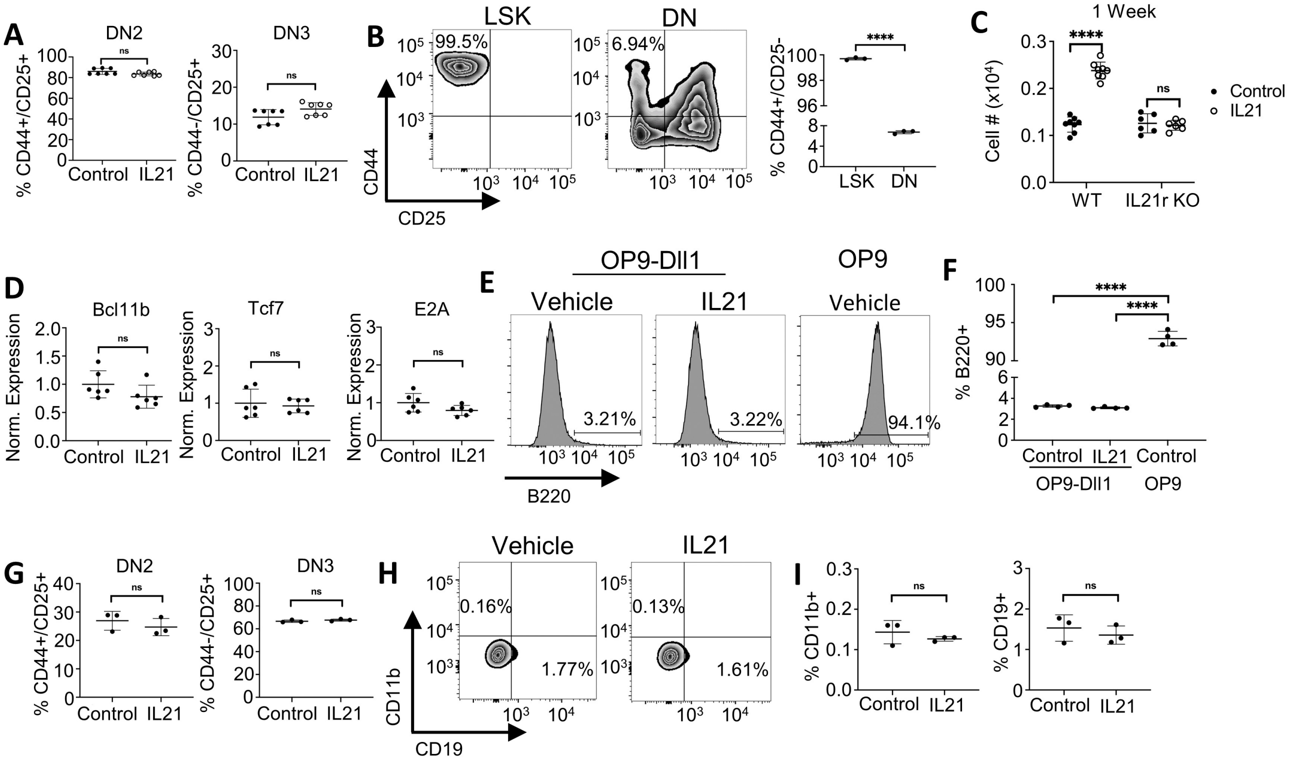

Supplemental Figure 4. Notch/IL21 co-cultures do not produce B cell or myeloid cells.

A. Percentage of 2-week OP9-Dll1 co-cultured LSK cells expressing thymocyte DN2 (CD44+/CD25+) or DN3 (CD44-/CD25+) markers (n=7), with or without IL21 treatment. Data generated using individual mice. **B**. (left) Representative flow plot for CD44 and CD25 on WT LSK cells and thymic CD4-/CD8- cells. Percentages of live cells are indicated. (right) Percentage of CD44+/CD25- WT LSK cells and thymic CD4-/CD8- cells. **C**. Cell count after 1-week coculture of WT (n=8) or IL21r KO (n=6) sorted LSK cells on OP9-Dll1 with or without IL21. Data generated using individual mice. **D**. Expression of Bcl11b, Tcf7, and E2A relative to GAPDH and normalized to control determined by qRT-PCR of mRNA from control and IL21 treated LSK 14-day cultures (n=6). Data generated using individual mice. **E**. Representative histogram of B220 staining on WT LSK cells cultured 2 weeks on OP9-Dll1 cells with or without IL21 or OP9 cells without IL21. **F**. Percentage of 2-week OP9 and OP9-Dll1 co-cultured LSK cells expressing B220 (n=4), with or without IL21 treatment. Data generated using individual mice. **G**. Percentage of cells expressing thymocyte DN2 (CD44+/CD25+) or DN3 (CD44-/CD25+) markers (n=3) on day 12 of culture on immobilized rhDll4 and rhVCAM-1, with or without IL21 treatment. Data generated using individual mice. **H**. Representative flow plot for CD11b and CD19 on WT LSK cells cultured 12 days on immobilized rhDll4 and rhVCAM-1 with or without IL21. Percentages of live cells are indicated. **I**. Percentage of cells expressing CD11b (left) or CD19 (right) (n=3) on day 12 of culture on immobilized rhDll4 and rhVCAM-1, with or without IL21 treatment. Data generated using individual mice. rh = Recombinant Human. ns $p>0.05$, **** $p<0.0001$. Statistical analysis performed using student t-test (**A-B,D,G,I**), One-way Anova (**F**) and Two-way Anova (**C**).

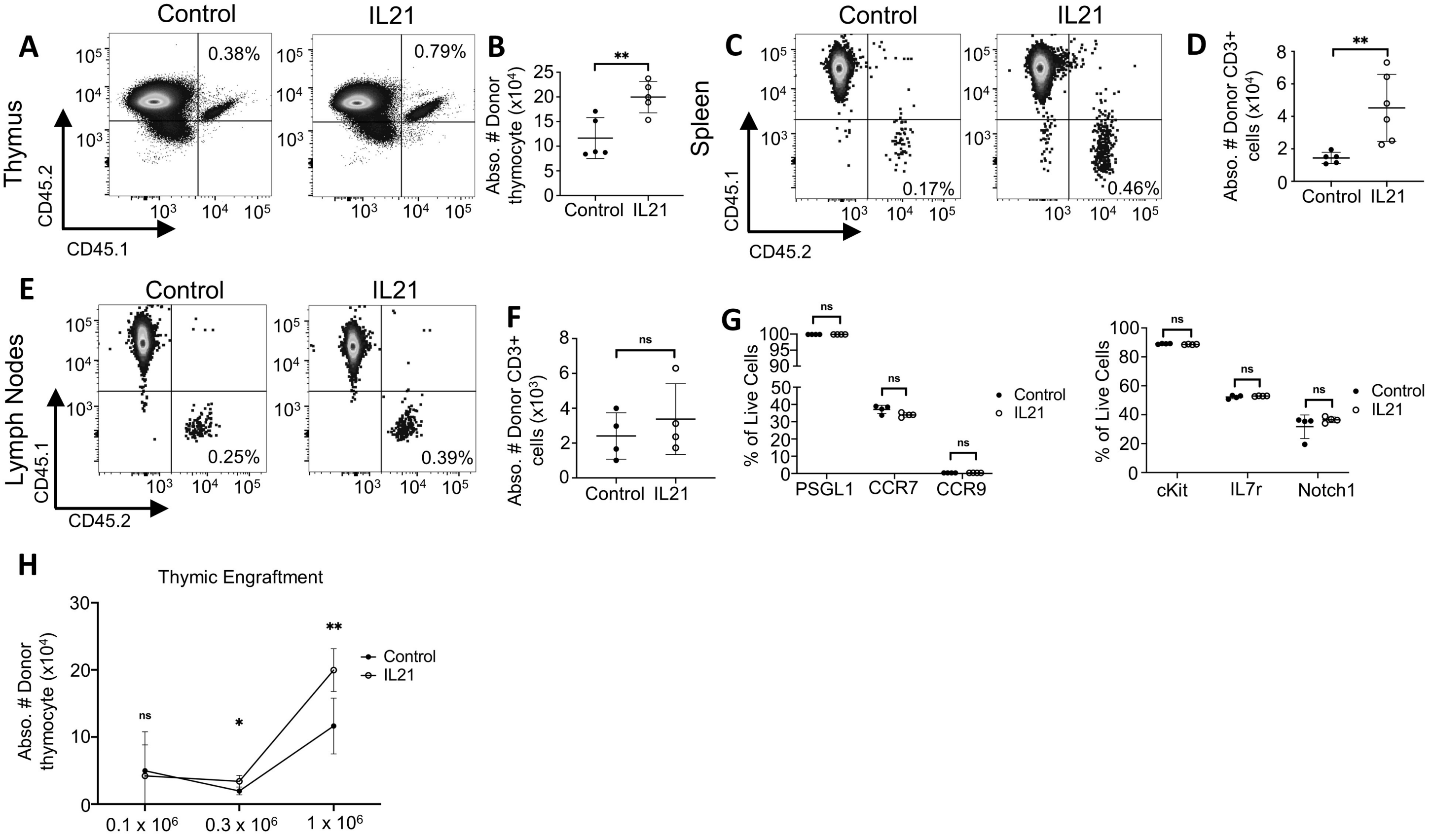

Supplemental Figure 5. Notch/IL21 primed T cell progenitors efficiently reconstitute irradiated recipients.

A. Representative flow plot for CD45.2+ (recipient) and CD45.1/.2+ (donor) thymocytes of 4.5 Gy irradiated mice transplanted with vehicle or IL21 treated cells from LSK/OP9-Dll1 cocultures, 1-week post-transplant with percentage of live cells shown. Equal numbers (1x10^{6}) of cells transplanted per condition. **B**. Absolute number of donor derived thymocytes 1-week post-transplantation (n=5). **C**. Representative flow plot for CD45.1+ (recipient) and CD45.2+ (donor) splenocytes of 4.5 Gy irradiated mice transplanted with vehicle or IL21 treated cells from LSK/OP9-Dll1 cocultures, 2-weeks post-transplant. Gated on CD3+. Equal numbers (3x10^{6}) of cells transplanted per condition. **D**. Absolute number of donor derived CD3+ splenocytes 2-weeks post-transplantation (Control n=5, IL21 n=6). **E**. Representative flow plot for CD45.1+ (recipient) and CD45.2+ (donor) lymph nodes cells of 4.5 Gy irradiated mice transplanted with vehicle or IL21 treated cells from LSK/OP9-Dll1 cocultures, 2-weeks post-transplant. Gated on CD3+. Equal numbers (3x10^{6}) of cells transplanted per condition. **F**. Absolute number of donor derived CD3+ lymph node cells 2-weeks post-transplantation (n=4). **G.** Percentage of cells expressing indicated homing (left) or differentiation (right) markers after 2-weeks culture on OP9-Dll1, with or without IL21 (n=4). **H**. Absolute number of donor derived thymocytes 1-week post-transplantation of 0.1x10^{6} (n=3), 0.3x10^{6} (n=4), or 1x10^{6} (n=5) vehicle or IL21 treated cells from LSK/OP9-Dll1 cocultures. ns $p>0.05$, * $p<0.05$, ** $p<0.01$. Statistical analysis performed using student t-test (**B-G**) and Two-way Anova (**H**).

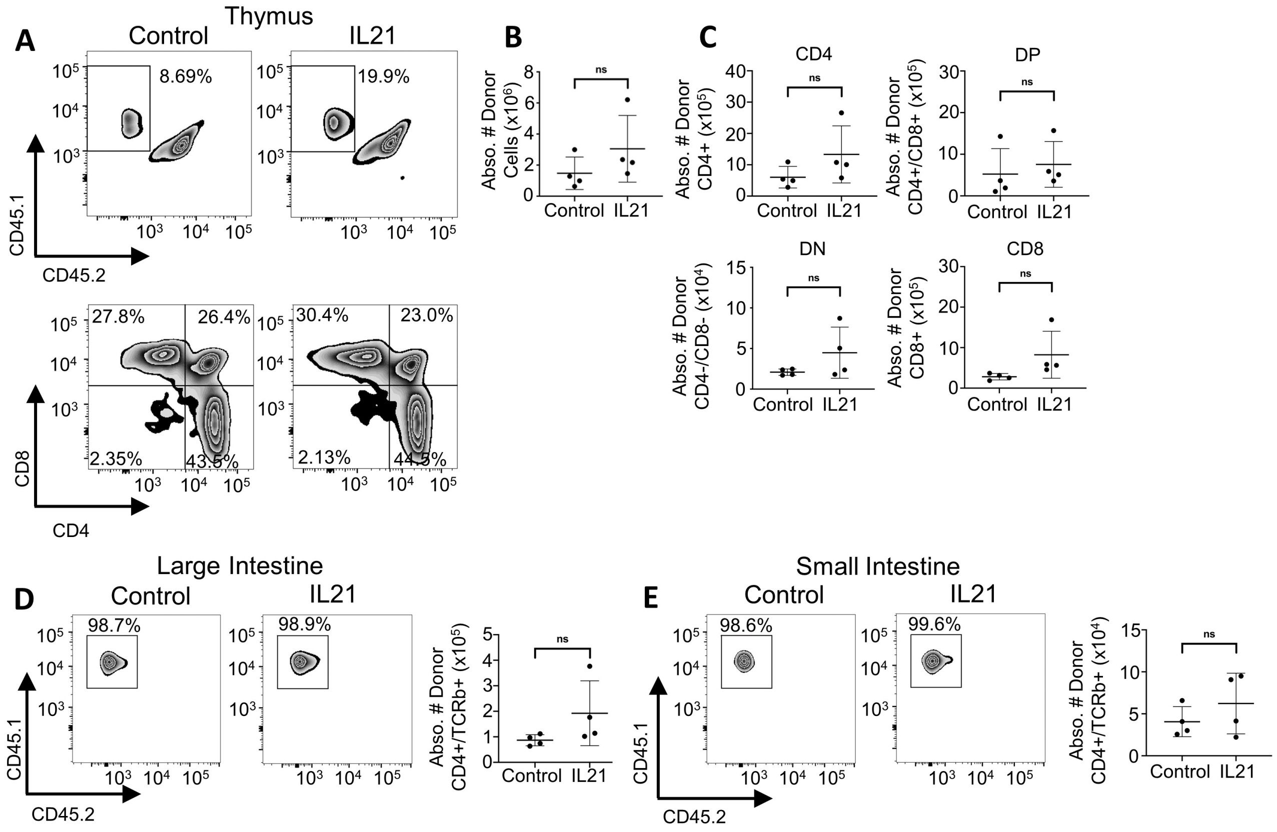

Supplemental Figure 6. Peripheral lymphoid reconstitution of *ex vivo* derived T cell progenitors in allogeneic transplant.

A. Representative flow plots for (Top) CD45.1+ (ex vivo derived) and CD45.1/.2+ (HSC derived) and (Bottom) CD4 and CD8 thymocytes of lethally irradiated LP/J recipient mice transplanted with control or IL21 treated LSK/OP9-Dll1 coculture derived T cell progenitors, 4-weeks post-transplant. Gated on (Top) CD3+/Ly9.1- and (bottom) CD3+/Ly9.1-/CD45.1+/CD45.2-. Equal numbers ($2x10^6$) of cells transplanted per condition. **B**. Absolute number of control (n=4) or IL21 (n=4) treated ex vivo co-culture derived cells in the thymus of lethally irradiated LP/J recipient mice. **C**. Absolute numbers of CD45.1+/CD45.2-/Ly9.1- (ex vivo derived) donor thymocyte subsets from control (n=4) or IL21 (n=4) treated co-cultures 4-weeks post-transplantation. DP = CD4+/CD8+, DN = CD4-/CD8-. **D**. (Left) Representative flow plot for donor T cells in Large Intestine, 1 month post transplant. Gated on MHCII-/TCRb+/CD4+/Ly9.1-. (Right) Absolute number of MHCII-/CD45.1+/CD45.2-/Ly9.1-/CD4+/TCRb+ T cells in Large Intestine from control (n=4) and IL21 (n=4) treated culture recipients. **E**. (Left) Representative flow plot for donor T cells in Small Intestine, 1 month post transplant. Gated on MHCII-/TCRb+/CD4+/Ly9.1-. (Right) Absolute number of MHCII-/CD45.1+/CD45.2-/Ly9.1-/CD4+/TCRb+ T cells in Small Intestine from control (n=4) and IL21 (n=4) treated culture recipients. ns $p>0.05$. Statistical analysis performed using student t-test (**B-E**).

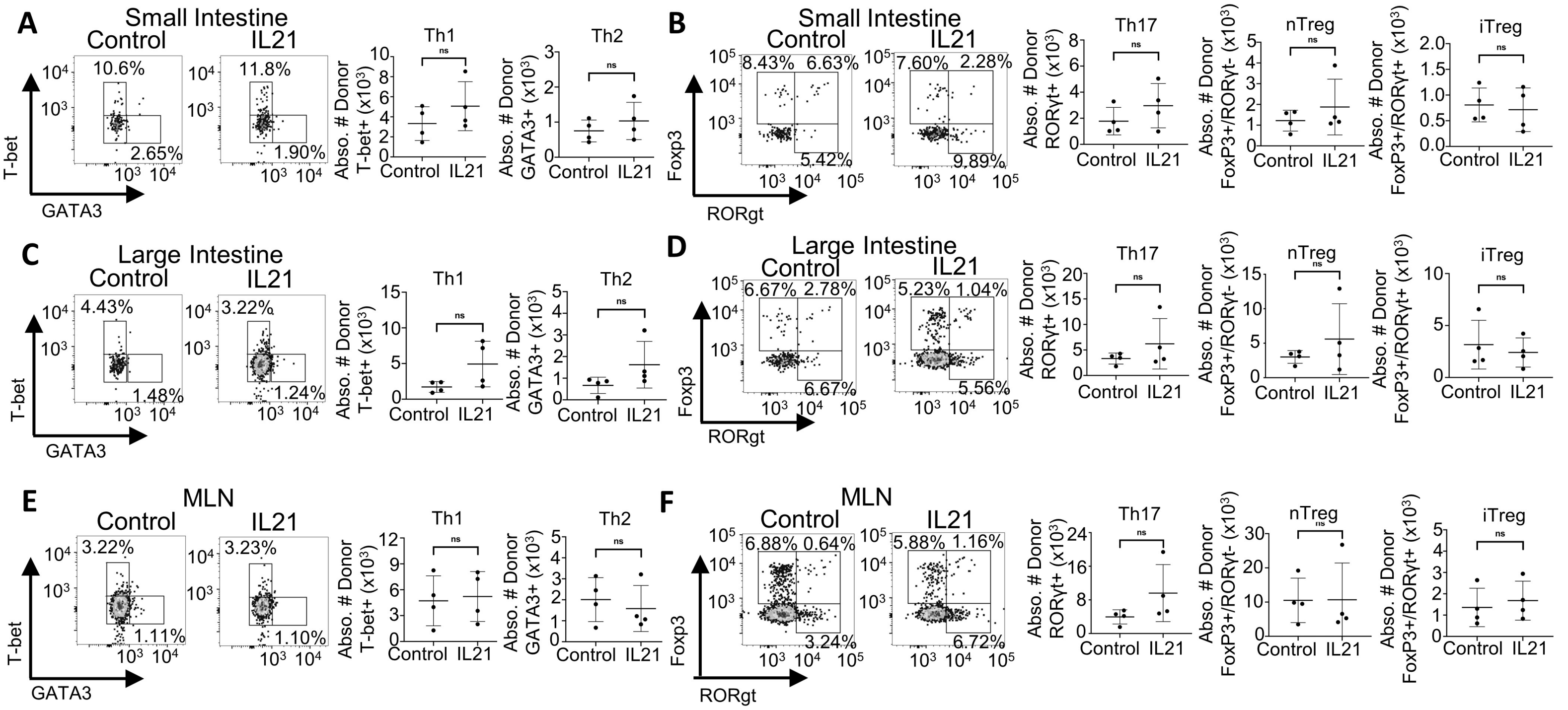

Supplemental Figure 7. IL21/Notch primed T cell progenitors function normally post-transplant.

A. (Left) Representative T-bet and GATA3 staining of donor T cells in SI, 1 month post transplant. Gated on MHCII-/Ly9.1-/CD4+/TCRb+/CD44+. (Right) Absolute number of donor T cell subtypes in SI of control (n=4) and IL21 (n=4) progenitor recipients, 1 month post transplant. **B**. (Left) Representative Foxp3 and RORγt staining of donor T cells in SI, 1 month post transplant. Gated on MHCII-/Ly9.1-/CD4+/TCRb+/CD44+. (Right) Absolute number of donor T cell subtypes in SI of control (n=4) and IL21 (n=4) progenitor recipients, 1 month post transplant. **C**. (Left) Representative T-bet and GATA3 staining of donor T cells in LI, 1 month post transplant. Gated on MHCII-/Ly9.1-/CD4+/TCRb+/CD44+. (Right) Absolute number of donor T cell subtypes in LI of control (n=4) and IL21 (n=4) progenitor recipients, 1 month post transplant. **D**. (Left) Representative Foxp3 and RORγt staining of donor T cells in LI, 1 month post transplant. Gated on MHCII-/Ly9.1-/CD4+/TCRb+/CD44+. (Right) Absolute number of donor T cell subtypes in LI of control (n=4) and IL21 (n=4) progenitor recipients, 1 month post transplant. **E**. (Left) Representative T-bet and GATA3 staining of donor T cells in MLN, 1 month post transplant. Gated on MHCII-/Ly9.1-/CD4+/TCRb+/CD44+. (Right) Absolute number of donor T cell subtypes in MLN of control (n=4) and IL21 (n=4) progenitor recipients, 1 month post transplant. **F**. (Left) Representative Foxp3 and RORγt staining of donor T cells in MLN, 1 month post transplant. Gated on MHCII-/Ly9.1-/CD4+/TCRb+/CD44+. (Right) Absolute number of donor T cell subtypes in MLN of control (n=4) and IL21 (n=4) progenitor recipients, 1 month post transplant. MLN = Mesenteric Lymph Nodes, LI = Large Intestine, SI = Small Intestine. ns p>0.05. Statistical analysis performed using student t-test (**A-F**).

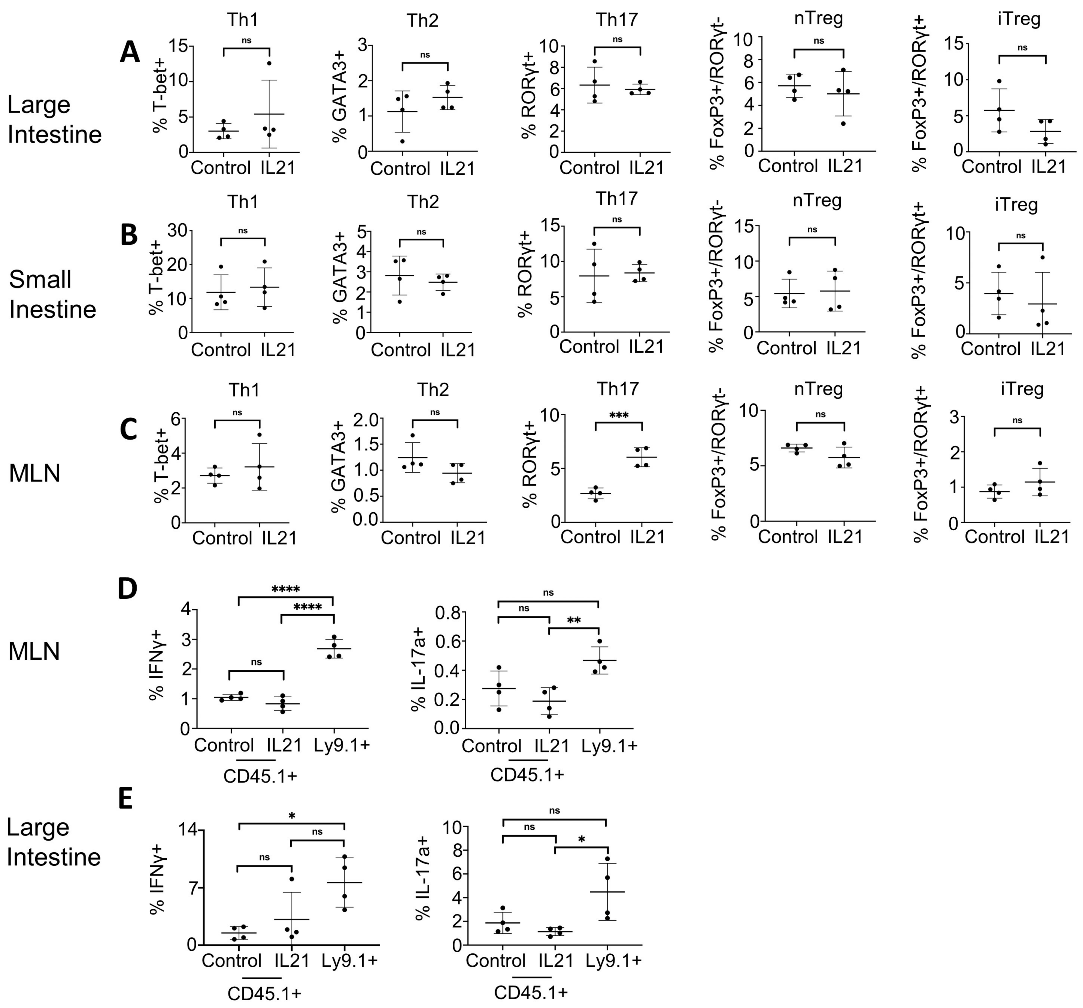

Supplemental Figure 8. IL21/Notch primed T cell progenitors differentiate normally post-transplant.

A. Percentages of donor derived CD4+/CD44+/TCRb+ T cells expressing indicated T cell subtype specific transcription factors from large intestine of control (n=4) and IL21 (n=4) treated culture recipients, 1-month post allogeneic transplant. **B**. Percentages of donor derived CD4+/CD44+/TCRb+ T cells expressing indicated T cell subtype specific transcription factors from small intestine of control (n=4) and IL21 (n=4) treated culture recipients, 1-month post allogeneic transplant. **C**. Percentages of donor derived CD4+/CD44+/TCRb+ T cells expressing indicated T cell subtype specific transcription factors from MLN of control (n=4) and IL21 (n=4) treated culture recipients, 1-month post allogeneic transplant. **D**. Percentages of CD4+/TCRb+ T cells expressing indicated cytokines from MLN of host Ly9.1+ (n=4), and control (n=4) and IL21 (n=4) *ex vivo* treated culture (CD45.1+) recipients, 1-month post allogeneic transplant. **E**. Percentages of CD4+/TCRb+ T cells expressing indicated cytokines from large intestine of host Ly9.1+ (n=4), and control (n=4) and IL21 (n=4) *ex vivo* treated culture (CD45.1+) recipients, 1-month post allogeneic transplant. MLN = Mesenteric Lymph Nodes. ns $p>0.05$, * $p<0.05$, ** $p<0.01$, *** $p<0.001$, **** $p<0.0001$. Statistical analysis performed using student t-test (**A-C**) and One-way Anova (**D-E**).
